# Tetra-*O*-methyl-nordihydroguaiaretic acid inhibits energy metabolism and synergistically induces anticancer effects with temozolomide on LN229 glioblastoma tumors implanted in mice while preventing obesity in normal mice that consume high-fat diets

**DOI:** 10.1371/journal.pone.0285536

**Published:** 2023-05-25

**Authors:** Kotohiko Kimura, Jong Ho Chun, Yu-Ling Lin, Yu-Chuan Liang, Tiffany L. B. Jackson, Ru Chih C. Huang

**Affiliations:** 1 Department of Biology, Johns Hopkins University, Baltimore, Maryland, United States of America; 2 Agricultural Biotechnology Research Center, Academia Sinica, Taipei, Taiwan, Republic of China; 3 Academician, Academia Sinica, Taipei, Taiwan, Republic of China; Facultad de Estudios Superiores Iztacala, Universidad Nacional Autónoma de México, MEXICO

## Abstract

Tetra-*O*-methyl-nordihydroguaiaretic acid (terameprocol; M_4_N), a global transcription inhibitor, in combination with a second anticancer drug induces strong tumoricidal activity and has the ability to suppress energy metabolism in cultured cancer cells. In this study, we showed that after continuous oral consumption of high-fat (HF) diets containing M_4_N, the M_4_N concentration in most of the organs in mice reached ~1 μM (the M_4_N concentration in intestines and fat pads was as high as 20–40 μM) and treatment with the combination of M_4_N with temozolomide (TMZ) suppressed glycolysis and the tricarboxylic acid cycle in LN229 human glioblastoma implanted in xenograft mice. Combination treatment of M_4_N with TMZ also reduced the levels of lactate dehydrogenase A (LDHA), a key enzyme for glycolysis; lactate, a product of LDHA-mediated enzymatic activity; nicotinamide phosphoribosyltransferase, a rate-limiting enzyme for nicotinamide adenine dinucleotide plus hydrogen (NADH)/NAD+ salvage pathway; and NAD+, a redox electron carrier essential for energy metabolism. It was also shown that M_4_N suppressed oxygen consumption in cultured LN229 cells, indicating that M_4_N inhibited oxidative phosphorylation. Treatment with M_4_N and TMZ also decreased the level of hypoxia-inducible factor 1A, a major regulator of LDHA, under hypoxic conditions. The ability of M_4_N to suppress energy metabolism resulted in induction of the stress-related proteins activating transcription factor 4 and cation transport regulator-like protein 1, and an increase in reactive oxygen species production. In addition, the combination treatment of M_4_N with TMZ reduced the levels of oncometabolites such as 2-hydroxyglutarate as well as the aforementioned lactate. M_4_N also induced methylidenesuccinic acid (itaconate), a macrophage-specific metabolite with anti-inflammatory activity, in tumor microenvironments. Meanwhile, the ability of M_4_N to suppress energy metabolism prevented obesity in mice consuming HF diets, indicating that M_4_N has beneficial effects on normal tissues. The dual ability of combination treatment with M_4_N to suppress both energy metabolism and oncometabolites shows that it is potentially an effective therapy for cancer.

## Introduction

Nordihydroguaiaretic acid (NDGA), a lignan, was first discovered in the creosote bush (*Larrea divaricata*), a desert medicinal plant [1]. NDGA has various biological properties, including antifungal and antimicrobial activities [2, 3]. However, at high concentrations, NDGA causes severe cytotoxicity. 3-*O*-methyl-nordihydroguaiaretic acid (Mal.4), a plant lignan derived from NDGA, is much less toxic than NDGA but can dose dependently suppress human immunodeficiency virus (HIV)-1 replication [4]. Electrophoretic mobility shift analysis has shown that Mal.4 prevents the eukaryotic transcription factor SP1 from binding to its cognate binding sites on the HIV long terminal repeat promoter. This is a likely mechanism by which Mal.4 inhibits transcription [4]. There are at least 12,000 Sp1 binding sites in the human genome, associated with genes involved in most cellular processes [5]. Therefore, Mal.4-related molecules can be considered a global transcription inhibitor. To anticipate possible clinical uses of lignans for treating viral diseases, methods have been established to synthesize preparative amounts of nine methylated NDGAs [6–9]. Among them, the fully methylated tetra-*O*-methyl-NDGA (terameprocol, M_4_N) is three times more active than the synthetic Mal.4 in anti-HIV assays [8, 9]. Although M_4_N can be easily synthesized from NDGA, it is also obtainable as a natural product [6].

Experiments using tissue cell culture have shown that M_4_N can induce G2 arrest in transformed mammalian cells [10]. Meanwhile, experiments using C57 black mice bearing C3 cell tumors (C3 cells were derived from C57 black mouse embryo cells transfected with full-length human papillomavirus 16 virus) showed that intralesional injections of M_4_N into tumors substantially decreased the tumor size with no detected host toxicity. Since then, our laboratory has performed many experiments using mouse xenograft tumor models to examine anticancer activity and the biochemical mechanisms of M_4_N [11–17]. However, the best evidence demonstrating the efficacy of M_4_N as an anticancer drug is a clinical trial of patients with oral squamous cell carcinoma conducted in India in the late 1990s [18]. The results of that trial suggested that M_4_N could potentially be an effective anticancer drug for oral cancers.

Since first proposed by Warburg, it has been well known for decades that cancer cells utilize energy metabolism differently from normal cells [19]. This suggests that it may be possible, by modulating energy metabolism, to create certain physiological environments that are unfavorable for cancer growth without drastically harming normal tissues. It was recently found that the manipulation of energy metabolism in immune cells affects the strength of immunity [20] and that certain metabolites secreted from cancer cells modulate tumor microenvironments (TMEs) and affect the activity of immune cells residing inside the TME [21, 22]. Thus, the crosstalk between tumor metabolism and immunity has become an important subject in anticancer therapy [23]. These new findings suggest that appropriately conditioned metabolic modulations might strengthen anticancer immunity as well.

Previously, we showed that M_4_N synergistically induced tumoricidal activity after combination treatments with second anticancer drugs using xenograft mouse models. We also showed that M_4_N suppressed energy metabolism in tissue culture cancer cells and that combination treatment with M_4_N induced the significant generation of reactive oxygen species, suggesting that this effect might be related to the anticancer activity of M_4_N [12]. In this study, we determined the effect of continuous M_4_N consumption on the energy metabolism of healthy mice by using weight change as an indicator and also by studying the systemic effect of the daily administration of combination treatment of M_4_N with temozolomide (TMZ) on energy metabolism in LN229 glioblastoma tumor cells implanted in mice.

## Materials and methods

### Reagents

M_4_N (10 mg/mL in CPE 25/30 formulation) was supplied by Erimos Pharmaceutical, LLC (Raleigh, NC, USA) [24]. Sorafenib, TMZ, etoposide, rapamycin, UCN-01, and cobalt chloride (CoCl_2_) were purchased from Millipore Sigma (St. Louis, MO, USA). The antibodies used in the experiments are described in the S1 Table.

### Cell culture

AsPC-1 pancreatic cancer, HepG2 hepatic cancer, and HeLa cervical cancer cell lines were purchased from American Type Culture Collection (Manassas, VA, USA). Green fluorescent protein (GFP)-labeled LN229 cells were purchased from Bioresource Collection and Research Center (Hsinchu City, Taiwan). All of these cell lines are of human origin. The HL-1 mouse heart cell line was a kind gift from Dr. Claycomb (LSU Health Science Centers, New Orleans, LA, USA) [25]. The cell culture conditions are available in the supplements.

### M_4_N/TMZ treatment *in vivo* and collection of LN229 tumor samples

Female BALB/c nude (nu/nu) mice aged 5 to 6 weeks were purchased from the National Laboratory Animal Center (Taipei City, Taiwan). Details about the animals are available in the supplements. BALB/c (nu/nu) mice were inoculated subcutaneously with 1×10^7^ GFP-labeled LN229 cells in 100 μL phosphate-buffered saline (PBS). When the average tumor mass reached 200–300 mm^3^, the tumor-bearing mice were randomly divided into four groups. Animals were treated with 150 mg/kg M_4_N and/or 2.5 mg/kg TMZ via daily oral administration. Treatment was stopped on day 25 for TMZ alone and M_4_N+TMZ and stopped on day 35 for the control and M_4_N alone. Vitamin E-Miglyol (EM) formulation was used as a vehicle for the drug treatment of LN229 tumor-bearing xenograft mice. Procedures for EM formulation were previously described [26]. Details about this formulation are available in the supplements. The EM solvent formulation was used as a control. Tumor measurements were recorded once per week using the Xenogen IVIS Imaging System (Xenogen, Alameda, CA, USA). After completion of the treatment schedule, the mice were sacrificed and the subcutaneous tumors were extracted.

### Drug treatments for HepG2 and AsPC1 tumor-bearing xenograft mice

T cell-deficient 8-week-old male nu/nu mice were obtained from Charles River Laboratories (Wilmington, MA, USA). These nude mice were used to study the effect of M_4_N treatment on xenotransplants of human-derived cancer, HepG2, and AsPC1 tumors. Implantation of tumors was performed as described in the supplements. The drug injection methods and schedules are available in the supplements.

### Assessment of weight in mice consuming high-fat diets containing M_4_N

Eight-week-old male C57BL|6J mice (000664; The Jackson Laboratory, Bar Harbor, ME, USA) were used to model diet-induced obesity. The details about the animals are available in the supplements. The mice were split into high-fat (HF) diet and HF diet containing the M_4_N drug (HFM) groups. HF mice were used as a control. The details about the ingredients of the food are available in the supplements. There were five mice in the HF and HFM groups. Each mouse had its own cage to track the amount of food that an individual mouse consumed. The experiment lasted a total of 8 weeks.

### Food consumption, weight measurements, and the estimation of M_4_N in the organs

The weight measurements were used to determine the effects of feeding mice an HFM diet. Food consumption was measured by the total grams of food given, minus the total grams of food left in the cages during each check-in date. Measurements were taken three times a week. Mice were weighed once a week, about the same time of day on each weighing date. The organs were collected on the final day of the experiments as described in the supplements. The content of M_4_N in the organs of mice that ate HFM diets were measured by a combination of thin layer chromatography (TLC) and high-performance liquid chromatography–tandem mass spectrometry (HPLC-MS/MS). The precise methods for the M_4_N assay are available in the supplements.

### Analysis of cell metabolites

LN229 tumor samples were collected as previously described. The samples were analyzed by Metabolon Inc. (Durham, NC, USA).

### Hypoxia experiments

For hypoxia experiments in LN229 cells, CoCl_2_ was used to mimic hypoxia conditions [27]. The cells were plated 2 days prior to the initiation of treatment, and the CoCl_2_ stock solution (100X in water) was added to the culture medium. At 16 h after treatment with CoCl_2_, the cell samples were collected. For hypoxia experiments for Hela cells, the cells were plated 1 day prior to the initiation of treatment. Exposure of cells to hypoxia was carried out in the PROOX C-Chamber with oxygen (O_2_) and carbon dioxide (CO_2_) levels modulated by the PROOX Model C21 Controller (BioSpherix, Lacona, NY, USA). The cells were exposed to 4% O_2_ for 10 h (moderate hypoxia) or exposed to six cycles of 1% O_2_ for 1 h followed by normoxia for 10 min, a combined total of 7 h of exposure (intermittent hypoxia). M_4_N stock solution was made in 100% dimethyl sulfoxide (DMSO). The final concentration of DMSO in the culture medium was 1.0%. Deferoxamine (DFO; Millipore Sigma) was added to the cultures as a 100X stock prepared in water (15 mM final concentration).

### Superoxide assay

The assay was performed using a mitochondrial superoxide detection kit (Abcam, Cambridge, UK) according to the manufacturer’s protocol. The cells were cultured in 96-well microwell dishes for 24 h and further incubated in 100 μL medium containing M_4_N (40 μM) and/or TMZ (30 μM) for an additional 2, 4, 24, and 48 h. Then 100 μL MitoROS 580 reagent, a fluorescence indicator for superoxide (a 500X stock solution diluted in the assay buffer), was added to each well. The cells were further incubated at 37°C for 60 min, and the fluorescence was measured at an excitation wavelength of 540 nm and emission wavelength of 590 nm (with a cutoff at 570 nm) with the Infinite M200 Microplate Reader (Tecan, Grödig, Austria).

### O_2_ consumption assay

The assay was performed using the O_2_ consumption rate assay kit (Cayman Chemicals, Ann Arbor, MI, USA). The cells were plated in 96-well black (clear bottom) tissue culture plates. The cells were treated with M_4_N dissolved in DMSO (final concentration of DMSO in the medium was 1%). After 24 h, O_2_ consumption was measured according to the company’s protocol. Briefly, the cells were treated with phosphorescent O_2_ probe and the medium inside the wells was covered with mineral oil to shield the O_2_ leaks. The intensity of fluorescence, which was an indicator for the amount of O_2_ consumption in the medium, was measured by the Infinite M200 Microplate Reader (Tecan, Grödig, Austria) at the wavelengths of 380/650 nm (Ex/Em), using the Time Resolved Fluorescence method.

### Western blot analyses

Western blotting was performed as previously described [12]. The detailed protocols are available in the supplements.

### RayBiotech Western blot analyses

This procedure was carried out for LN229 tumors removed from xenograft mice. The tissue samples were prepared according to the company’s protocol (RayBiotech, Peachtree Corner, GA, USA). Western blotting was performed by RayBiotech.

### Northern blot analyses

Total RNA was extracted from cells with Trizol Reagent (Invitrogen, Carlsbad, CA, USA) and isolated according to the manufacturer’s protocol. Northern hybridization was carried out as previously described [11]. The detailed protocols are available in the supplements.

### MTT (3-(4,5-dimethylthiazol-2-yl)-2,5-diphenyl tetrazolium bromide) assay

The MTT assay was performed as previously described [10]. The detailed protocols are available in the supplements.

### Trypan blue exclusion assay

For the Trypan blue exclusion assay, the cells were washed once with phosphate-buffered saline and resuspended in the buffer. One part of the resuspended cell solution was mixed with one part of 0.4% Trypan blue solution (Millipore Sigma). After 15 min, the numbers of cells with and without staining were counted. The percentage of stained cells to the total cell number (i.e., with + without staining) was calculated.

### Statistical analyses

The statistical analyses were performed with the Student’s *t*-test (SigmaPlot; SPSS Inc., UK). The synergy between two drugs in their activities was assessed by CompuSyn software (ComboSyn Inc., NJ, USA).

## Results

### M_4_N synergistically induces strong tumoricidal activity in combination

**Fig 1:**
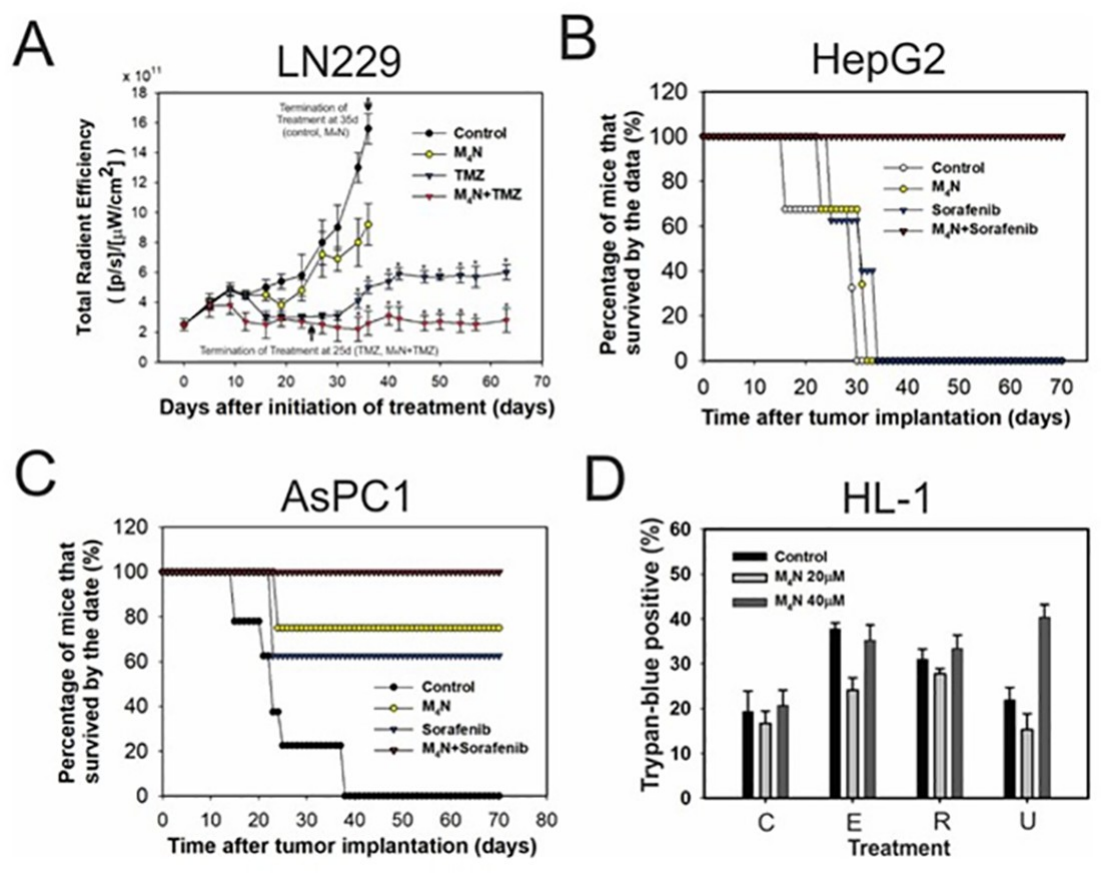
Daily combination treatments of M_4_N with secondary anticancer drugs synergistically induced strong tumoricidal activity. **(A)** Effects of combination treatment of M_4_N with TMZ on tumor growth in nu/nu mice implanted with glioblastoma LN229 cells. LN229 xenograft mice (N = 5/group) were treated with or without M_4_N by daily oral administration for 35 days. Meanwhile, another set of LN229 xenograft mice (N = 5/group) were treated with TMZ only or M_4_N+TMZ by daily oral administration for 25 days, and the examination of tumor growth was continued even after the termination of drug treatments. The tumor volumes of M_4_N, TMZ, and M_4_N+TMZ groups were significantly different compared to the control group after day 23 (*p<0.05). The bars indicate standard deviations. **(B-C)** The effects of combination treatments of M_4_N with sorafenib on the survival rates of nu/nu mice (N = 5) implanted with various tumors were examined (A-B). Drugs were administered daily via intravenous tail vein injection (B-C). **(B)** AsPC-1 pancreatic tumors. **(C)** HepG2 hepatic tumors. **(D)** Effect of combination treatments of M_4_N with etoposide, rapamycin, or UCN-01 on HL-1 mouse heart cells. Cell death was examined by the Trypan blue exclusion assay in HL-1 cells treated with combination treatments for 24 h. The concentrations of M_4_N are shown in the figure. The concentrations of etoposide, rapamycin, and UCN-01 were 10, 20, and 5 μM.

Our previous research showed that M_4_N has the ability to reduce tumor growth; however, the drug treatment was not as effective in shrinking the tumor when the drug concentration was insufficient [11–17, 26, 28–30]. To boost the anticancer activity of M_4_N, the addition of a second anticancer drug was introduced. The synergistic anticancer effects of M_4_N combination treatments, using various drugs, have been demonstrated in many human cancer cells in both tissue cultures and mouse xenograft experiments [12–14]. Fig 1A–1C shows examples demonstrating the effectiveness of anticancer combination therapy with M_4_N in human brain (LN229), pancreatic (AsPC-1), and hepatic (HepG2) tumors. These data showed that M_4_N combination treatment with TMZ or sorafenib increased the survival rate of cancer-bearing mice, whereas single drug treatments with M_4_N, TMZ, or sorafenib failed to have a significant effect on survival rate. To determine the effect of combination treatment with M_4_N in non-cancerous cells, the cytocidal activity of M_4_N in combination with etoposide, rapamycin, or UCN-01 in HL-1 mouse heart cells [25] was examined with the Trypan blue exclusion assay (Fig 1D). HL-1 cells represent a slow-growing cardiac myocyte cell line that can be repeatedly passaged and still maintain a cardiac-specific phenotype [25]. The data showed that M_4_N did not synergistically induce cell death with etoposide, rapamycin, or UCN-01 in HL-1 cells (Fig 1D) unlike many cancer cells that were treated with combination treatments of M_4_N with various second anticancer drugs including etoposide, rapamycin, and UCN-01 [11–17].

### M_4_N prevents obesity in mice that consumed an HF diet

**Fig 2:**
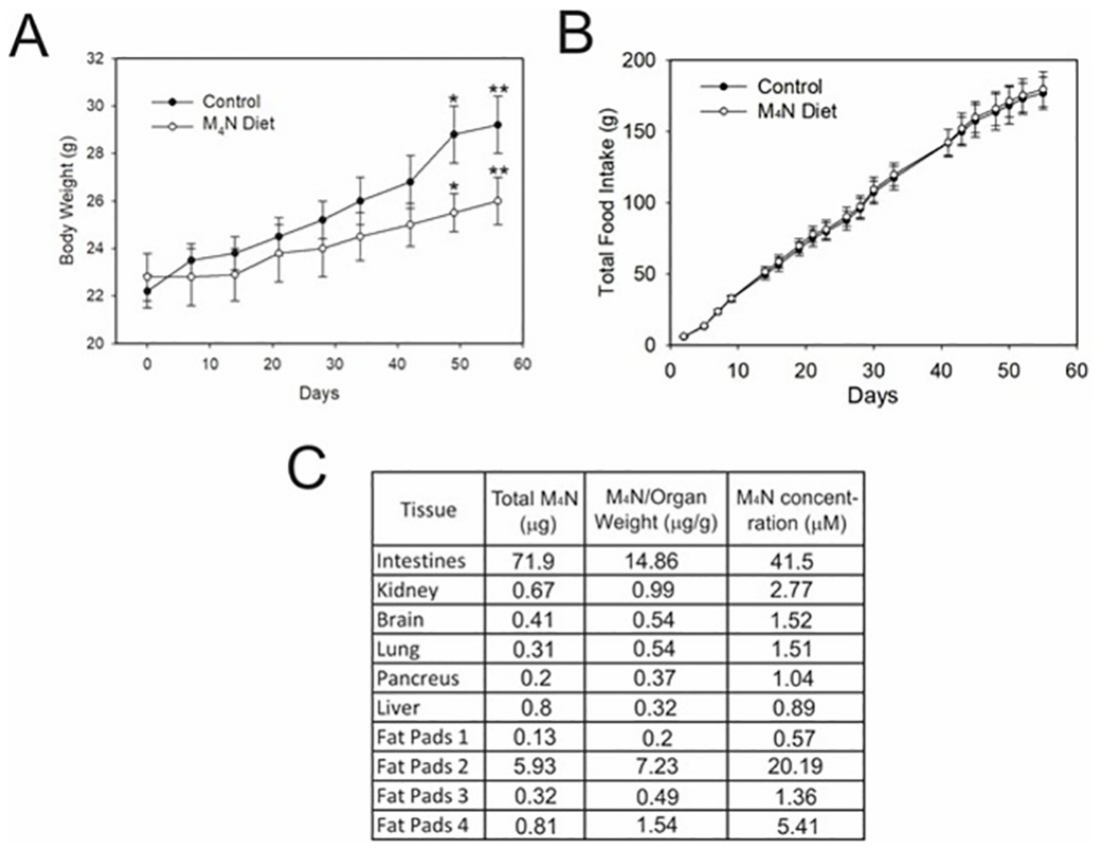
M_4_N prevents obesity in mice consuming HF diets. **(A)** M_4_N prevented obesity in mice that consumed HF diets. Male C57BL/6J mice (N = 5) consumed HF diets containing corn oil, either HFM diets (6.83 mg M_4_N/g food) or food without M_4_N. The body weights were measured periodically. The bars indicate standard deviations. The points designated by ‘*’ or ‘**’ indicate that the differences between the control and the group whose food contained M_4_N were statistically significant by the Student’s *t*-test (p<0.05). **(B)** Total food intake of male C57BL/6J mice (N = 5) during long-term systemic oral administration of either HFM or control diets were measured periodically. The bars indicate standard deviations. Food intake did not significantly differ between the control mice and the mice that consumed HFM diets. **(C)** A C57BL/6J mouse consumed food containing 25 mg/mL M_4_N (6.83 mg M_4_N/g food) for 16 weeks and the amount of M_4_N in the tissues was measured. M_4_N concentrations were calculated based on the amount of M_4_N extracted from the dry tissue samples, and then were correlated back to the wet weight of the original tissue sample. The standard curve for these calculations ranged from 0.1 to 10 ng/mL.

In a previous study, we showed that M_4_N suppressed energy metabolism in tissue culture cancer cells [12]. Here, we systemically evaluated how much M_4_N treatment affected energy metabolism by examining how much the consumption of diets containing M_4_N affected the body weight of laboratory mice. Fig 2A shows the body weight of normal mice that consumed HFM diets compared to those that consumed HF diets without M_4_N. Mice that consumed HFM diets did not gain as much weight as those that consumed food without M_4_N, indicating that supplementing diets with M_4_N prevented mice from becoming overweight even when eating HF diets [31]. Fig 2B shows that both groups consumed almost the same amount of food. The amount of M_4_N in various organs of the mice that ate HFM diets was measured. Fig 2C shows the M_4_N concentration in various organs. Drug concentration was highest in the gastrointestinal tract, which was directly exposed to the drug during food absorption. Drug concentration was also high in fat tissues, where the lipophilic nature of M_4_N likely facilitated its retention.

### M_4_N combination treatments suppress glycolysis and the tricarboxylic acid cycle via suppression of lactate dehydrogenase and nicotinamide phosphoribosyltransferase

**Fig 3:**
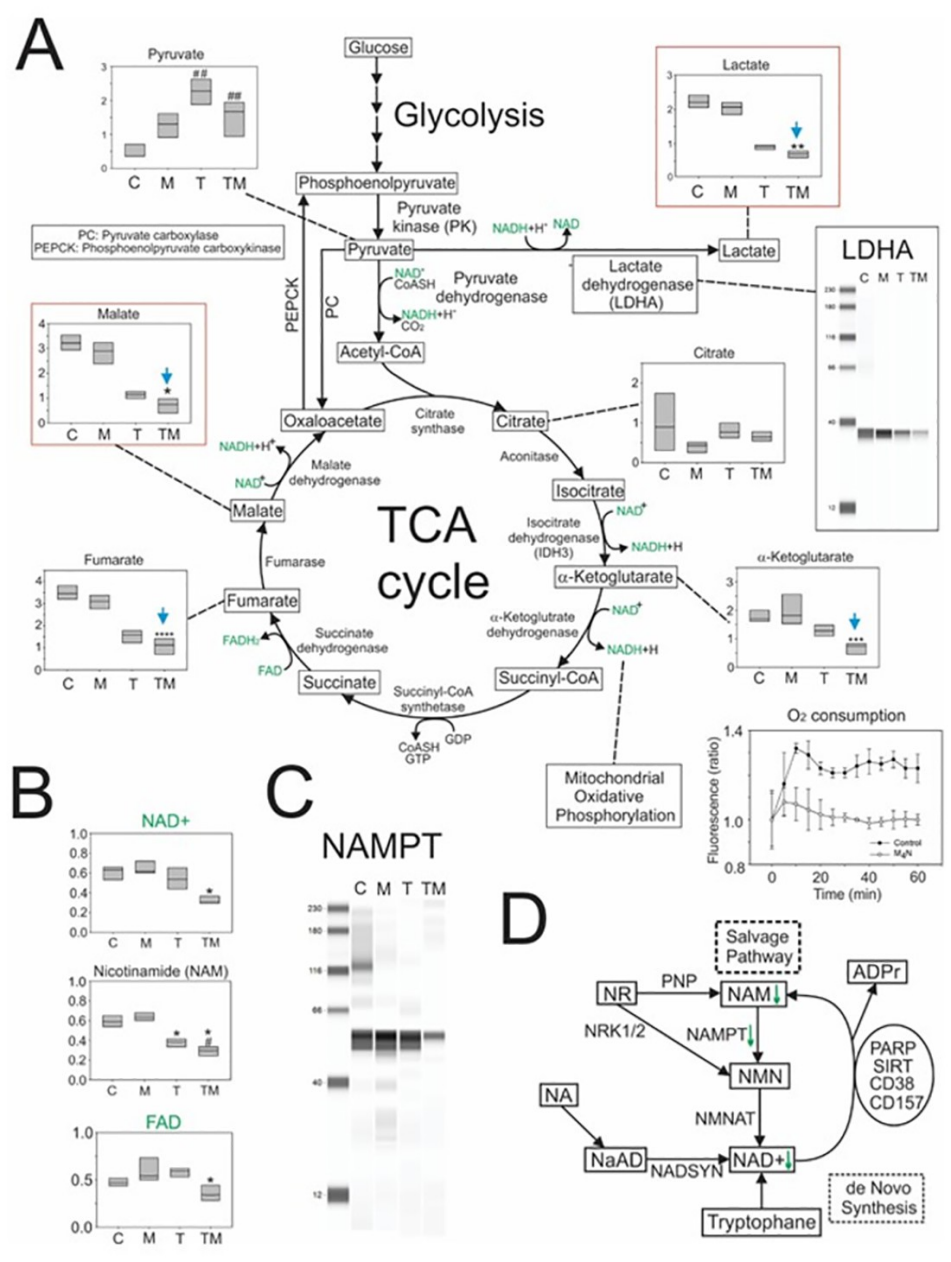
Effects of M_4_N combination treatment on glycolysis, the TCA cycle, the salvage pathway of nicotinamide adenine dinucleotide synthesis, and the production of flavin adenine dinucleotide. **(A)** The box figures show the amounts of metabolites in the treated tumors on an arbitrary scale. The upper edge of each box represents a limit of the upper quartile, whereas the lower edge represents a limit of the lower quartile. The line in the middle of each box represents a median value. Asterisks show that the difference between the LN229 tumors treated with TMZ (T) alone and those with TMZ+M_4_N (TM) were statistically significant by the Student’s *t*-test (*p<0.05, **p<0.02, ***p<0.01, and ****p<0.1). Sharp marks show that the difference between the LN229 tumors treated with vehicle alone and either those with TMZ (T) alone or those with TM were statistically significant by the Student’s *t*-test (##p<0.01). The amounts of lactate, α-ketoglutarate, fumarate, and malate were smaller in the TM than T group (indicated by blue downward arrows). Right inlet figure: TM combination treatments suppressed the expression of LDHA in LN229 tumors transplanted in xenograft mice. Lower right inlet figure: M_4_N suppressed the O_2_ consumption of LN229 cells. LN229 cells were treated with M_4_N (30 μM) for 24 h. O_2_ consumption, which is an indicator of the activity of mitochondrial oxidative phosphorylation, was measured by an O_2_ consumption rate assay kit (Cayman Chemicals, Ann Arbor, MI, USA). When the concentration of O_2_ is lower, the intensity of the fluorescence becomes stronger. The bars in the figure indicate the standard deviations. There were statistically significant differences between the control and M_4_N-treated cells at all the time points later than 10 min by the Student’s *t*-test (p<0.05). **(B)** Effect of M_4_N (M) and/or T on the intracellular contents of NAD^+^, nicotinamide, and FAD. The data points are from the tumors of five mice. One asterisk (*) indicates that there was a significant difference between either control and T, or control and TM by the Student’s *t*-test (p<1%), whereas one sharp mark (#) indicates that there was a significant difference between T and TM by the Student’s *t*-test (p<5%). The upper edge of each box represents a limit of the upper quartile, whereas the lower edge represents a limit of the lower quartile. The line in the middle of each box represents a median value. **(C)** Effect of M and/or T on the expression of NAMPT. **(D)** Schematic showing the effect of the combination treatment of TM on the mechanisms of NAD^+^ synthesis. See S2 Table for metabolite abbreviations.

To understand how M_4_N affects the metabolism of tumors inside the body, human LN229 glioblastoma cells were implanted in xenograft mice, and these mice were treated with vehicle only, M_4_N only, TMZ only, or M_4_N+TMZ in combination, and then the metabolites in the tumors were compared among these mice. Metabolite analysis of the LN229 tumors showed that while TMZ only treatment suppressed the content of lactate, an end product of glycolysis, and that of malate, an end product of the TCA cycle, to some extent, M_4_N+TMZ combination treatment significantly further suppressed the contents of both lactate and malate (Fig 3A). The level of LDHA, a key glycolysis-related enzyme that catalyzes the enzymatic reaction to convert pyruvate to lactate, was suppressed by TMZ to some extent and was markedly suppressed by M_4_N+TMZ combination treatment (Fig 3A, a right inlet figure). These data indicate that the suppression of glycolysis by M_4_N+TMZ combination treatments occurs by reducing LDHA levels. Previously, it was shown that M_4_N induced mitochondrial membrane hyperpolarization [12], which indicate that M_4_N can significantly modulate the physiological properties of mitochondria. Other than glycolysis, ATP is generated in the mitochondria by oxidative phosphorylation, which requires O_2_ for its reactions [32]. The O_2_ consumption assay showed that M_4_N suppressed the O_2_ consumption of LN229 culture cells (Fig 3A), indicating that M_4_N suppresses ATP generation by oxidative phosphorylation.

Nicotinamide adenine dinucleotide (NAD+) is deeply involved in energy metabolism for all kinds of nutrients [33]. NAD+ is produced by either *de novo* synthesis or the NAD+ salvage pathway. The metabolite assay for LN229 tumors transplanted in xenograft mice showed that M_4_N+TMZ combination treatment significantly suppressed the contents of NAD+ and nicotinamide, both of which are metabolites of the NAD+ salvage pathway (Fig 3B). Western blot analysis showed that M_4_N+TMZ combination treatment significantly reduced the expression of nicotinamide phosphoribosyltransferase (NAMPT), a rate-limiting enzyme of the salvage pathway (Fig 3C). A schematic figure about the mechanisms of NAD+ production, including the *de novo* pathway and the salvage pathway, is shown in Fig 3D. These data indicated overall that M_4_N+TMZ combination treatment suppressed the NAD+ salvage pathway. M_4_N+TMZ combination treatment suppressed the levels of both NAD+ and flavin adenine dinucleotide (FAD) (Fig 3B). The reduction of NAD+ and FAD levels should reduce the performance of the TCA cycle since NAD+ and FAD are required for many reactions in the TCA cycle (Fig 3A).

### M_4_N promotes the degradation of hypoxia-inducible factor 1 subunit alpha

Hypoxia-inducible factor 1 subunit alpha (HIF1A) is very important for the progression of cancer since regions of hypoxia are commonly associated with rapidly growing solid tumors as they outgrow their blood supply, and the response to this O_2_ starvation is the stabilization of HIF1A [34, 35]. HIF1A also plays a significant role in regulating glycolysis [19, 34, 35]. Under hypoxic conditions, HIF1A is markedly induced and increases the expression of many hypoxia-responsive genes including LDHA, which is the most important enzyme for regulating glycolysis [34, 35]. Since M_4_N+TMZ combination treatment markedly suppressed the levels of LDHA in LN229 tumors implanted in mice (Fig 3A), the levels of HIF1A in LN229 cells treated with M_4_N and/or TMZ were examined.

**Fig 4:**
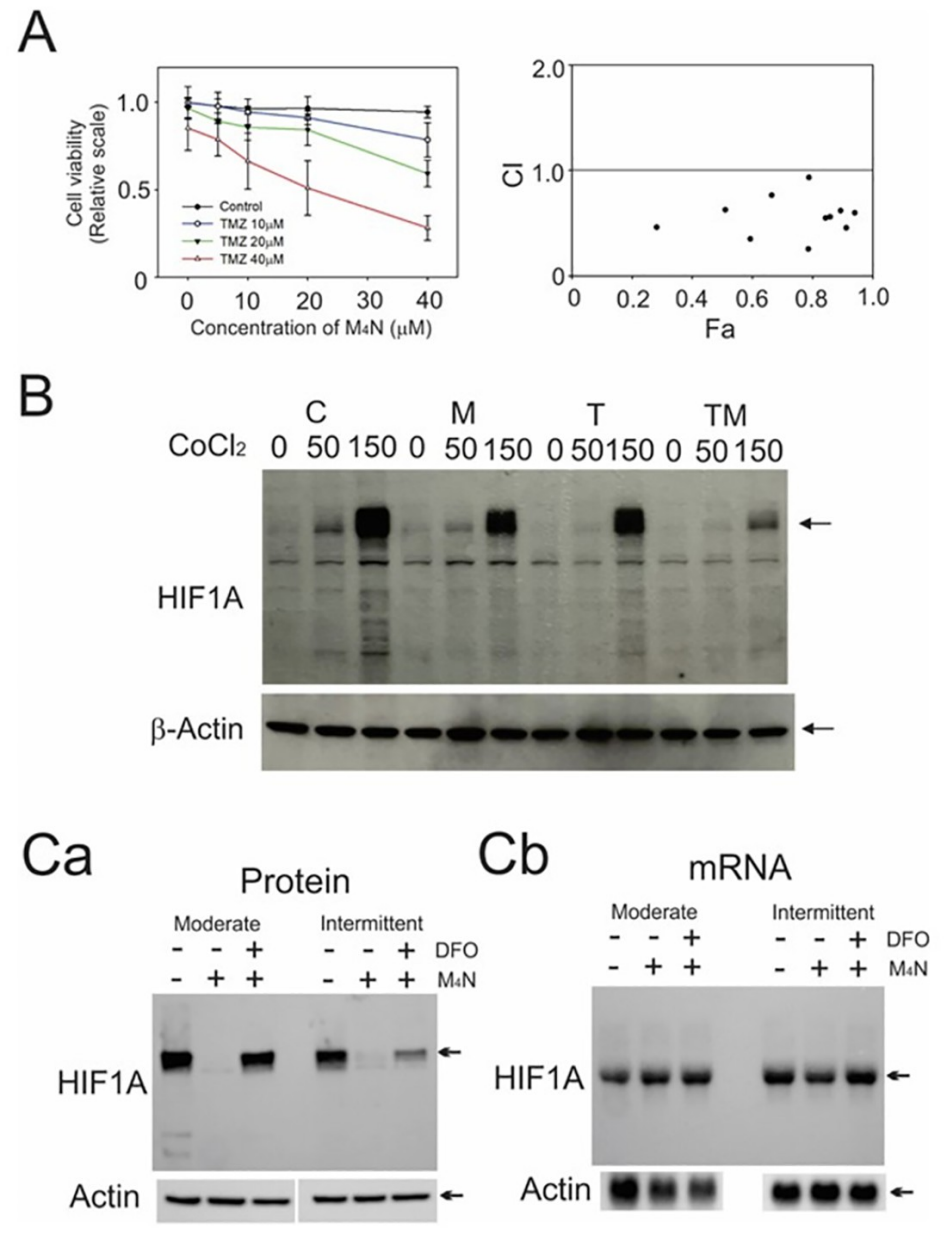
M_4_N treatment promoted the degradation of HIF1A. **(A) Left panel:** M_4_N+TMZ (TM) combination treatment synergistically reduced the viability of cultured LN229 cells. LN229 cells were treated with M_4_N (M) and/or TMZ (T) at the concentrations indicated in the figure. Cell viability was examined by the MTT assay at 72 h after treatment. The bars indicate standard deviations (N = 8). Right panel: A combination index (CI) plot obtained by the CompuSyn software for the experiment shown in the left panel. A CI less than 1.0 indicates that there is a synergy between two drugs. **(B)** M or TM combination treatment reduced HIF1A contents in cultured LN229 cells. LN229 cells were treated with M (40 μM) and/or T (30 μM) in the presence or absence of 50 or 150 μM CoCl_2_, which mimics hypoxic conditions. The cells were collected at 16 h after the treatment and the contents of HIF1A were examined by Western blotting. β-actin was used as a control. The arrows indicate the bands for HIF1A and β-actin. Control–vehicle only (C), M, T, and TM. **(C)** M_4_N treatment induces the rapid degradation of HIF1A in cultured HeLa cervical cancer cells during moderate and intermittent hypoxia. HeLa cells were exposed to either moderate hypoxia (4.0% O_2_) for 10 h or intermittent hypoxia (see Materials and Methods) for 7 h in the presence or absence of 60 μM M_4_N, and HIF1A protein levels were determined by Western blot analysis (**Ca**). The extent of HIF1A protein loss due to PHD-dependent proteasome-mediated degradation was ascertained by co-treatment of the cells with DFO (150 μM) (**Ca**). β-actin was used as a control (**Ca**). Levels of *HIF1A* mRNA were assessed by Northern blot analysis (**Cb**). The blot was re-probed with a β-actin cDNA probe to control for gel loading and transfer (**Cb**).

The MTT assay (Fig 4A) showed that M_4_N combination treatment synergistically reduced the viability of LN229 cells in the same manner as in many other cultured cancer cells [12]. Using cultured LN229 cells and CoCl_2_ to mimic hypoxia [27], the effect of M_4_N on the amount of HIF1A was examined. Western blotting (Fig 4B) showed that CoCl2, as expected, increased HIF1A contents in a dose-dependent manner and that M_4_N treatment either with or without TMZ reduced the amount of HIF1A in the presence of CoCl_2_.

Additionally, the effect of M_4_N on intracellular contents of HIF1A under hypoxic conditions was examined in cultured human Hela cervical cancer cells. HIF1A protein was undetectable under normoxia and induced under either moderate or intermittent hypoxia. However, the protein levels of HIF1A were dramatically decreased after treatment with M_4_N (Fig 4Ca). Meanwhile Northern blotting revealed no significant difference in *HIF1A* mRNA levels from M_4_N treated or untreated cells exposed to hypoxia (Fig 4Cb). Therefore, M_4_N must exert its negative effect on HIF1A either by inhibiting translation or more likely by promoting or sustaining degradation of the HIF1A protein during hypoxia. The hydroxylation and subsequent degradation of HIF1A in hypoxia are mediated by the prolyl hydroxylase domain (PHD) O_2_ sensors [36]. DFO [37] inhibits the degradation by chelating the iron required for degradation processes. If M_4_N promotes hypoxic degradation of HIF1A via a PHD-dependent mechanism, the addition of DFO should reverse the process and restore its stability. When cells exposed to hypoxia were treated with M_4_N and DFO concomitantly, the normal hypoxic levels of HIF1A protein were restored (Fig 4Ca). These results suggest that M_4_N promotes HIF1A under hypoxic conditions via a PHD-dependent degradation. These experiments (Fig 4C) using cultured Hela cells overall supported the conclusions from the experiments using LN229 cells and CoCl_2_ (Fig 4B).

### M_4_N induction of a stress-related signal transduction mechanism

**Fig 5:**
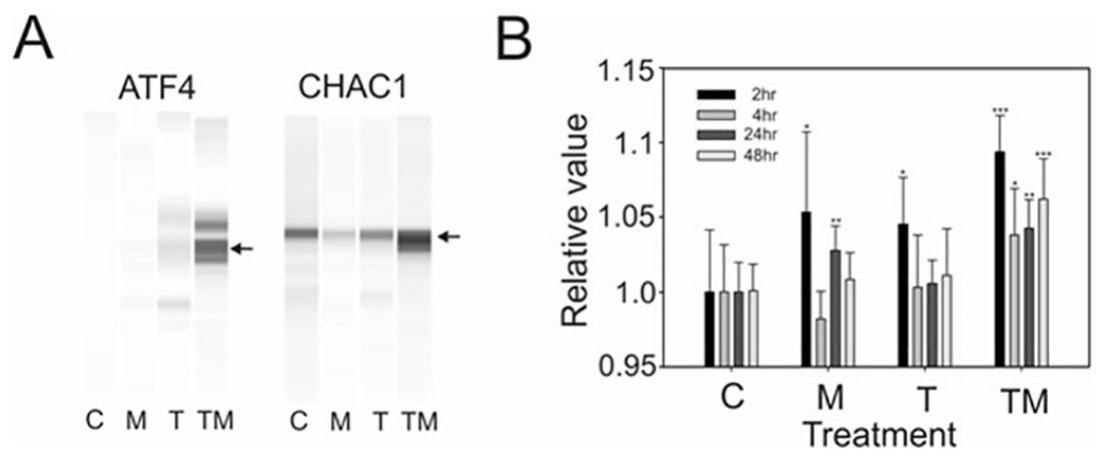
M_4_N+TMZ synergistically induced stress-related proteins, ATF4, and CHAC1 in tumors from LN229 xenograft mice and synergistically generated superoxide in LN229 tissue culture cells. **(A)** Two stress-related proteins, ATF4 and CHAC1, were induced in LN229 tumors implanted in nu/nu mice treated orally with M_4_N (M) and TMZ (T) for 25 days. The proteins were detected by Western blotting. **(B)** TMZ+M_4_N (TM) combination treatment synergistically induced the production of superoxide in cultured LN229 cells. The superoxide assay using MitoROS 580 dyes was performed in LN229 cells treated with M (40 μM) and/or T (30 μM) for 2, 4, 24, or 48 h. Control—vehicle only (C), M, T, or TM. The bars indicate standard deviations (N = 8). Asterisks show that the difference between the control and the LN229 tumors treated with M, T, or TM were statistically significant by the Student’s *t*-test (*p<0.05, **p<0.01, and ***p<0.001).

Since the energy metabolism in LN229 tumors implanted in nu/nu mice was suppressed by the combination treatment of M_4_N and TMZ (Fig 3), we determined whether M_4_N combination treatments could induce severe stress in LN229 tumors implanted in mice. The western blots (Fig 5A) showed that M_4_N+TMZ combination treatment markedly induced stress proteins, activating transcription factor 4 (ATF4), and ChaC glutathione-specific gamma-glutamylcyclotransferase 1 (CHAC1), in LN229 tumors. It is well known that endoplasmic (ER) stress and generation of reactive oxygen species (ROS) are closely associated [38]. The results of our experiment suggested that M_4_N+TMZ combination treatment might induce more ROS than either M_4_N or TMZ treatment alone. These data (Fig 5B) in fact showed that M_4_N and TMZ synergistically induced superoxide [39], a ROS predominantly produced in the electron transport chain in the mitochondria of LN229 cells. The data also suggested that the mitochondrial electron transport chain was not working efficiently in LN229 cells treated with either M_4_N alone or M_4_N+TMZ since superoxide induced an energetic loss during mitochondrial oxidative phosphorylation [40].

### M_4_N combination treatment reduces the levels of two oncometabolites, lactate and 2-hydroxyglutarate, in cancer cells whereas M_4_N alone treatment increases the levels of methylidenesuccinic acid (itaconate), a macrophage-specific metabolite, in the TME

**Fig 6:**
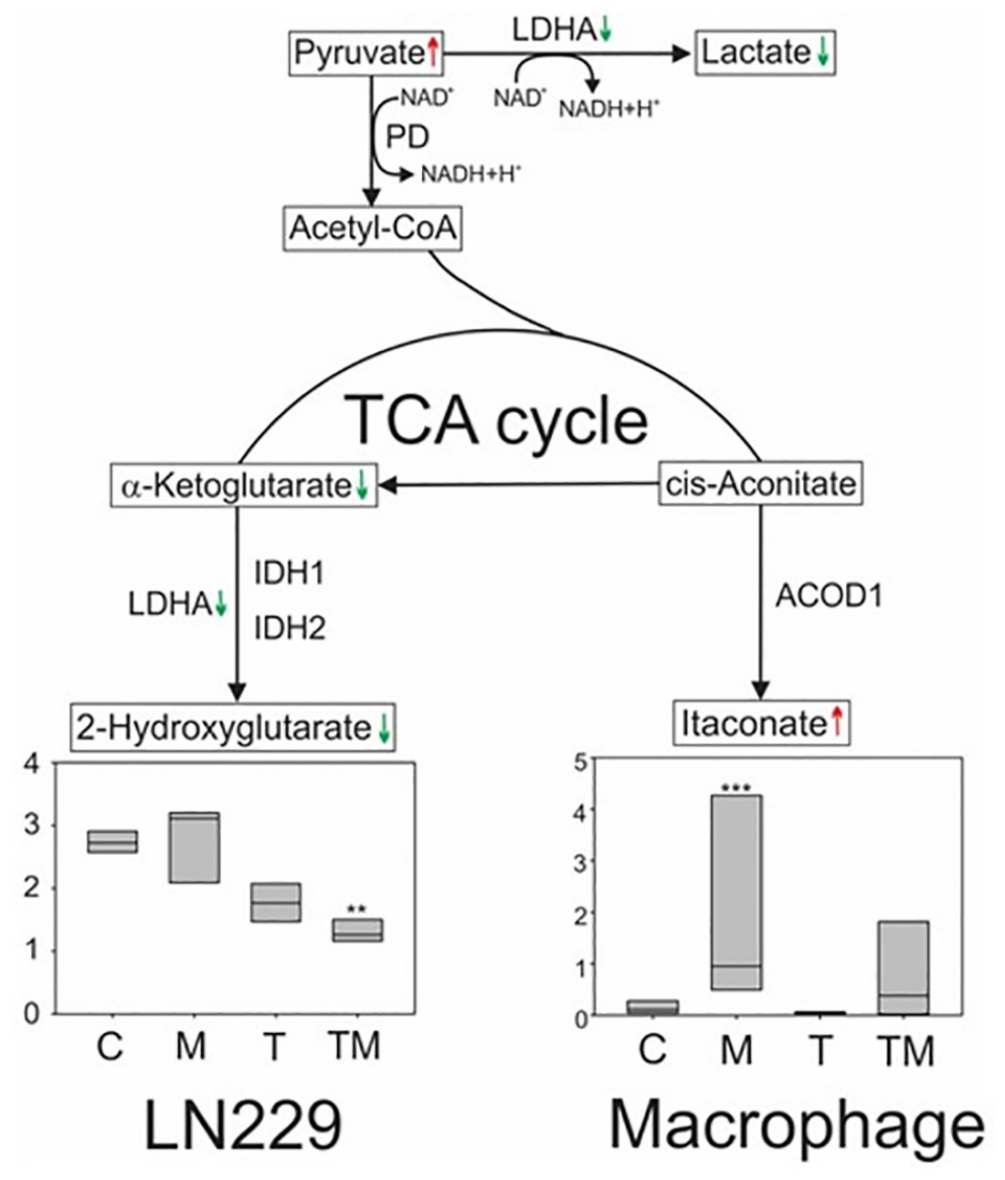
Lactate and 2-HG were suppressed in LN229 tumors from xenograft mice treated with M_4_N and TMZ for 25 days, and itaconate was induced in macrophages infiltrated in these tumors. The box figures showed the amounts of metabolites in the tumors (which included both LN229 cancer cells and their associated cells such as macrophages infiltrated in the tumors) treated with drug on an arbitrary scale. The difference in amount of 2-hydroxyglutarate (2-HE) between the tumors treated with TMZ (T) alone and those with TMZ+M_4_N (TM) was statistically significant by the Student’s *t*-test (**p<0.05). The difference in amount of itaconate between the control and the tumor treated with M_4_N (M) was statistically significant by the Student’s *t*-test (***p<0.01). The data points are from tumors of five mice. The upper edge of each box represents a limit of the upper quartile, whereas the lower edge represents a limit of the lower quartile. The line in the middle of each box represents a median value. The green downward arrows indicate that the contents in lactate, α-ketoglutarate, and LDHA were reduced by TM, compared with T alone. The red upward arrows indicate that the content of itaconate was increased by M, compared with the control or that the content of pyruvate was increased by T alone and TM than the control. Itaconate was produced from macrophage-related cells only (indicated by the designation ‘macrophage’), whereas 2-HE and lactate were produced by any cells including LN229 cells (indicated by the designation ‘LN229’). See S2 Table for enzyme abbreviations.

In xenograft mice implanted with LN229 tumors, M_4_N significantly changed the contents of lactate, 2-hydroxyglutarate (2-HG), and itaconate, which all modulated immunity and inflammation in the TME [22, 41] (Fig 6). As shown in Fig 3, M_4_N+TMZ combination treatment significantly reduced the content of lactate. Second, M_4_N+TMZ combination treatment significantly reduced the contents of 2-HG as well (Fig 6). 2-HG exists in two different chemical forms, (*R*)-2-hydroxyglutarate (*R*-2-HG) and (*S*)-2-hydroxyglutarate (*S*-2-HG) [42, 43]. The data for 2-HG represent the total amount of both (*R*) and (*S*) forms of this molecule. It has been shown that 2-HG as well as lactate can suppress the functions of immune cells in the TME after they are excreted from cancer cells [21, 22, 42, 43]. Third, M_4_N significantly induced itaconate in LN229 tumors (Fig 6). Itaconate is a direct product of citrate [41]. Since itaconate is produced predominately in macrophage-related cells [41], the itaconate detected in LN229 tumor samples was probably derived from macrophages infiltrating the LN229 tumors. Itaconate secreted from macrophages alleviates inflammation reactions in the cells residing in the vicinity of these macrophages [41].

## Discussion

M_4_N, a global transcription inhibitor, in combination with a second anticancer drug has been shown to induce strong tumoricidal activity when administered daily to tumor-bearing xenograft mice (Fig 1) [11–17]. When HL-1 cells were treated with M_4_N in combination with second anticancer drugs, the treatments did not induce cell death synergistically (Fig 1D). This was in contrast with many other observations using various cancer cells where M_4_N consistently induced cell death synergistically with various second anticancer drugs [11–17]. Although HL-1 cells are not totally normal cells, they retain most of the characters of normal cells [25]. This suggests that the cytotoxicity of M_4_N’s combination treatments probably work better against cancerous rather than non-cancerous cells. Since HL-1 cells grow slowly [25], the activity of M_4_N to suppress energy metabolism probably does not work efficiently against HL-1 cells unlike cancer cells, which grow fast and require a great deal of nutrients to survive. When healthy normal mice consumed M_4_N-containing diets daily for weeks, M_4_N accumulated and M_4_N concentrations reached at least 1 μM in nearly all organs (Fig 2C). Fulciniti et al. [44] showed that only 1 μM M_4_N inhibited the growth of multiple myeloma cells. Thus, effective M_4_N concentration is achievable by continuous oral administration regardless of where the cancer is located.

Previously, it was shown that M_4_N suppressed energy metabolism in cultured cancer cells [12]. In this study, we estimated the impact of continuous consumption of M_4_N-containing diets on energy metabolism of mice by measuring the weight changes of these mice. The results showed that M_4_N prevented obesity in mice that consumed HF diets (Fig 2A and 2B), suggesting that M_4_N affected the energy metabolism of the whole body of these mice when the drug was systemically administered. It was shown that M_4_N combination treatment with either TMZ or etoposide reduced the contents of long-chain acylcarnitines while either maintaining or increasing contents of long-chain fatty acids in LN229 cells implanted in xenograft mice or in LNCaP cultured cells, respectively (S1 Fig). This indicated that M_4_N combination treatment suppressed the conversion of fatty acids into acylcarnitines, an essential biochemical reaction for the initiation of β-oxidation of lipid catabolism, which probably hindered the utilization of fats as energy sources. Since the concentrations of M_4_N in the fat tissues reached as high as 20 μM after continuous M_4_N consumption (Fig 2C), M_4_N should be able to affect fat metabolism efficiently. Lee et al. [45] showed that NDGA prevented HF diet-induced fatty liver in obese mice, which suggested that lignans in general might suppress fat metabolism. These data (Fig 2A and 2B) indicated that M_4_N could potentially be used as a drug to control obesity.

Metabolism is an important part of tumorigenesis as well as the progression of cancer [46–48]. M_4_N combination treatment with TMZ suppressed both the conversion from pyruvate to lactate by LDHA, the last enzymatic reaction of glycolysis, and the TCA cycle in LN229 tumors (Fig 3A). The combination treatment of M_4_N with either etoposide or rapamycin also strongly suppressed the contents of lactate and malate (the last metabolite in the TCA cycle) in LNCaP prostate cancer cells or L428 Hodgkin’s lymphoma cells [11] (S2A and S2B Fig), supporting the data for LN229 tumors (Fig 3A). NAD+ is an essential coenzyme that participates in glycolysis, the TCA cycle, β-oxidation, and oxidative phosphorylation [33]. Thus, the drugs that can suppress the production of NAD+ should be able to greatly impact energy metabolism. In fact, inhibitors for NAMPT, a rate-limiting enzyme for the NAD+ salvage pathway, impair energy metabolism through disruption of specific metabolic pathways and increase energetic stress [49]. In addition, the expression of *NAMPT* is strongly correlated with the aggressiveness and stemness of cancer [50, 51]. The current study showed that M_4_N+TMZ combination treatment reduced the content of NAMPT protein (Fig 3C) and suppressed NAD+ content in LN229 tumors in xenograft mice (Fig 3B). This indicated that this combination treatment could act as an NAMPT inhibitor. In addition, M_4_N+TMZ combination treatment also suppressed the content of FAD (Fig 3B). These data may explain how M_4_N combination treatments suppressed the activity of the TCA cycle, which requires many NADH and FADH_2_ molecules for its performance (Fig 3A). Promoter computer analysis showed that there were numerous SP1 binding elements in the vicinity of the transcription start site of *NAMPT* gene promoter (S2C Fig), which suggested that M_4_N combination treatment reduced NAMPT content via its inhibitory effect on the SP1 transcription factor binding to the *NAMPT* gene promoter [5]. It was shown that M_4_N suppressed the O_2_ consumption of cultured LN229 cells (Fig 3; lower right inlet), which indicated that oxidative phosphorylation in the mitochondria was inhibited by M_4_N. Previously it was shown that M_4_N treatment induces mitochondrial membrane hyperpolarization [12]. These data indicate that M_4_N modulates the mitochondria and suppresses the mitochondrial electron transport system [32]. The overall metabolite data showed that the combination treatments with M_4_N suppressed both glycolysis and the mitochondrial electron transport system, two major mechanisms that generate ATPs, as well as the TCA cycle which is a major source of the building materials for amino acids, nucleic acids, and other important biochemical compounds, and should incur strong stress on cancer cells, which requires a lot of biological energy and materials to proliferate and survive.

The results (Fig 4Ca and 4Cb) showed that M_4_N facilitated the degradation of HIF1A, so that M_4_N reduced the amount of HIF1A under either moderate or intermittent hypoxia in HeLa cells. Normal tissue O_2_ levels vary within and among organs, but typically fall in a range of 3–9% [37]. Thus, moderate hypoxia (4% O_2_) is quite attainable even under normal physiological conditions in humans. Using LN229 cells and CoCl_2_, which mimics hypoxia, this study also showed that M_4_N and M_4_N+TMZ treatments reduced the amount of HIF1A (Fig 4B). HIF1A facilitates cancer development through the promotion of glycolysis via activation of LDHA, a key glycolysis-related enzyme [19, 20, 34, 35], and the content of LDHA was markedly reduced by M_4_N+TMZ combination treatment (Fig 3A) [47]. These data suggested that M_4_N suppressed glycolysis by reducing the contents of HIF1A and then LDHA (Fig 3A).

This study showed that M_4_N+TMZ combination treatment significantly increased the contents of stress-related proteins ATF4 and CHAC1 in LN229 tumors (Fig 5A). As previously shown [12], deep RNA-sequencing data demonstrated that M_4_N treatment induced numerous stress-related genes in human LNCaP prostatic, AsPC1 pancreatic, and L428 leukemic cancer cells [52] (S3A Fig), which was also confirmed by Western blot analysis of L428 cells (S3B Fig). *ATF4*, *ATF3*, DNA damage inducible transcript 3 (*DDIT3*), and *CHAC1* are among the stress-related genes induced by M_4_N that constitute a signaling cascade pathway starting with *ATF4* and ending with *CHAC1* (ATF4-ATF3-DDIT3-CHAC1 mechanism) (S3A and S3B Fig) [53]. In addition, other stress-inducible proteins such as CCAAT-enhancer-binding protein, sestrin 2, asparagine synthetase, TSC22 domain family member 3, and protein phosphatase 1 regulatory subunit 15A were also induced by M_4_N treatment (S3A, S3B Fig).

The direct causes of M_4_N-mediated induction of multiple stress-related genes are not clearly understood (Fig 5A, S3A and S3B Fig). It was shown that M_4_N suppressed energy metabolism in various metabolic pathways (Figs 3 and 4, and S1 Fig). This should trigger an intense ER stress response in tumors that proliferate uncontrollably and demand a great deal of nutrients. ER stress causes the production of ROS [38, 54]. In fact, it was shown that M_4_N with a second anticancer drug synergistically induced ROS [12] (Fig 5B and S3C Fig). ROS causes necrosis when its cytosolic concentration becomes significantly high [55], which likely explains how M_4_N combination treatments induce cell death more efficiently than single-drug treatments. A possible mechanism for this synergistic induction of ROS production by M_4_N combination treatments involves the mitochondria. M_4_N combination treatments suppress cytosolic contents of NAD+ and FAD (Fig 3B), and thus should inhibit mitochondrial electron transports. Impairment of the mitochondrial electron transport system by NAD+ depletion causes a great increase in ROS production and induces cell death [56], suggesting that M_4_N combination treatments can induce ROS production by reducing NAD+ contents. It was also previously shown that M_4_N induces hyperpolarization of the mitochondrial membrane potential [12, 57], which supports the involvement of mitochondria in M_4_N-related cell death.

M_4_N+TMZ combination treatment significantly reduced the contents of lactate and 2-HG (the total amount of *R-* and *S*-2-HG) in LN229 tumors (Fig 6). Lactate and R-2-HG suppress T cell-mediated immunity [58–60] and *S*-2-HG mitigates redox stress under hypoxia [42, 61]. Thus M_4_N+TMZ combination treatment can increase anticancer immunity and induce stress via reduction of these oncometabolites. Lactate is produced from pyruvate via LDHA (Fig 6). R-2-HG is produced from α-ketoglutarate via isocitrate dehydrogenase 1 (IDH1) or IDH2 [62], whereas S-2-HG is produced from α-ketoglutarate via LDHA (Fig 6). It was shown that M_4_N+TMZ combination treatment reduced the contents of both LDHA and α-ketoglutarate (Fig 3A), which is likely the reason that the contents of lactate and 2-HG were reduced by M_4_N+TMZ treatment. In addition, it was shown that M_4_N increased the content of itaconate in LN229 tumors (Fig 6). Since itaconate is formed only in macrophage-related cells [41], the detected itaconate in LN229 tumors was probably derived from macrophage-related cells infiltrating these tumors. Itaconate secreted from macrophages alleviates inflammation and inhibit some glycolysis- and TCA cycle-related enzymes in neighboring cells including LN229 tumor cells [41, 63, 64]. Inflammation aggravates the rampant chromosomal abnormalities often associated with metastatic cancer cells [65]. Thus, the anti-inflammatory activity of M_4_N that induces itaconate might reduce the aggressive nature of certain cancer cells.

## Conclusion

**Fig 7:**
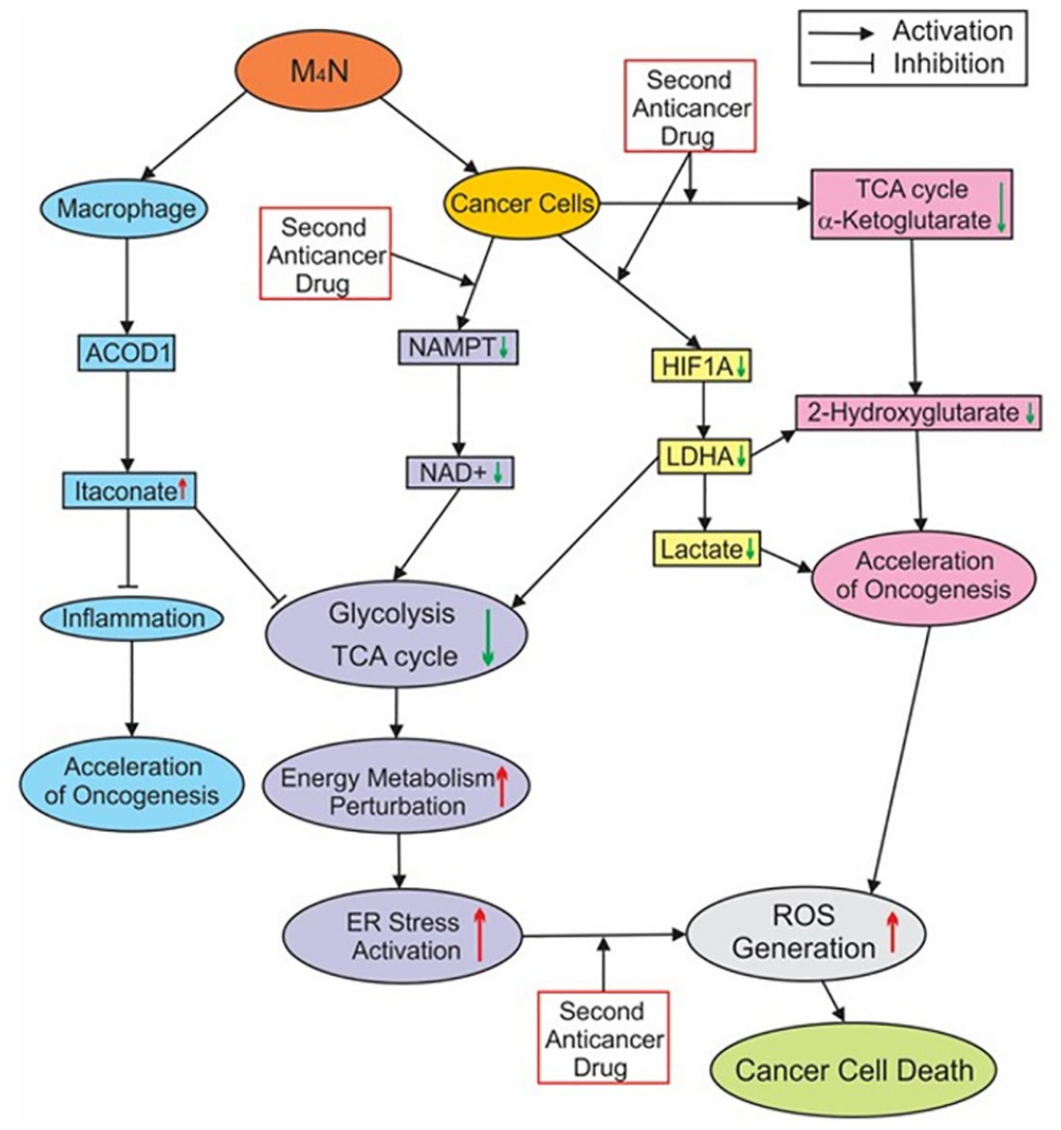
A presumptive schematic about the mechanism underlying the ability of M_4_N combination treatment to induce tumoricidal activity. The arrow accompanying each item indicates the effect of M_4_N (upward arrow in red: augmentation by M_4_N; downward arrow in green: suppression by M_4_N). See S2 Table for metabolite abbreviations.

Fig 7 summarizes the current understanding of the mechanisms underlying the anticancer activity of M_4_N. M_4_N suppresses energy metabolism in glycolysis and the TCA cycle (Fig 3A and S2A and S2B Fig) by modulating the cellular contents of various proteins, including HIF1A, LDHA, and NAMPT (Figs 3 and 4). The reduced energy metabolism activates ER stress mechanisms (Fig 5A, S3A and S3B Fig). ER stress increases ROS (Fig 5B and S3C Fig) particularly when second anticancer drugs are combined with M_4_N. The reduction of NAD+/FAD contents (Fig 3B) impairs the activity of the mitochondrial electron transport system [12], which induces ROS as well. When intracellular contents of ROS are exceedingly great, the anticancer effect is induced. Meanwhile, M_4_N combination treatments reduce the contents of both lactate and 2-HG (Fig 6), which suppress the functions of immune cells in the TME [21, 22, 58–60]. Thus, M_4_N combination treatments overall enhance anticancer immunity. In addition, M_4_N induces itaconate production in macrophages, which infiltrate into tumors (Fig 6) [41]. Secreted itaconate inhibits glycolysis and the TCA cycle and accelerates oncogenesis in the cancer cells that reside in the neighborhood of macrophages [39, 63–65].

Our findings show that an effective approach to possible complete remission of human cancer is through M_4_N combination treatment with selective anticancer drugs. This approach to anticancer therapy has three important characteristics. First, since efficient energy metabolism is crucial for the survival of any cancer cells, M_4_N combination treatment should be potentially applicable for cancers of heterogeneous origin [66]. Second, the ability of M_4_N combination treatment to reduce the contents of oncometabolites such as lactate or 2-HG showed that M_4_N combination treatment induced anticancer activity through its effects on immune-related metabolism as well as energy metabolism. Since these oncometabolites modulate the activities of multiple components of anticancer immunity regardless of differences in tumor antigens [58–60], M_4_N combination treatment should be applicable for cancers of heterogeneous origins from this perspective as well. Third, in addition to its potential usage as an anticancer drug, M_4_N can be a drug to prevent obesity for healthy individuals due to its activity to control energy metabolism.

## Supporting information

S1 Table A list of antibodies used for Western blotting. (TIF)

S2 Table Abbreviation list. (TIF)

S1 Fig M_4_N combination treatments suppressed long-chain acylcarnitine contents. (TIF)

S2 Fig Supplemental data about the effect of M_4_N on energy metabolism. (TIF)

S3 Fig M_4_N induced stress-related genes and synergistically generated reactive oxygen species in combination treatments. (TIF)

S1 File Expanded material and methods. (DOCX)

S2 File Supplemental materials and methods. (DOCX)
