# Supplementary figures and images for: Tetra-O-methyl-nordihydroguaiaretic acid inhibits energy metabolism and synergistically induces anticancer effects with temozolomide on LN229 glioblastoma tumors implanted in mice while preventing obesity in normal mice that consume high-fat diets
Source: PLoS One. 2023 May 25;18(5):e0285536. doi: 10.1371/journal.pone.0285536 (PMC10212158; doi:10.1371/journal.pone.0285536)

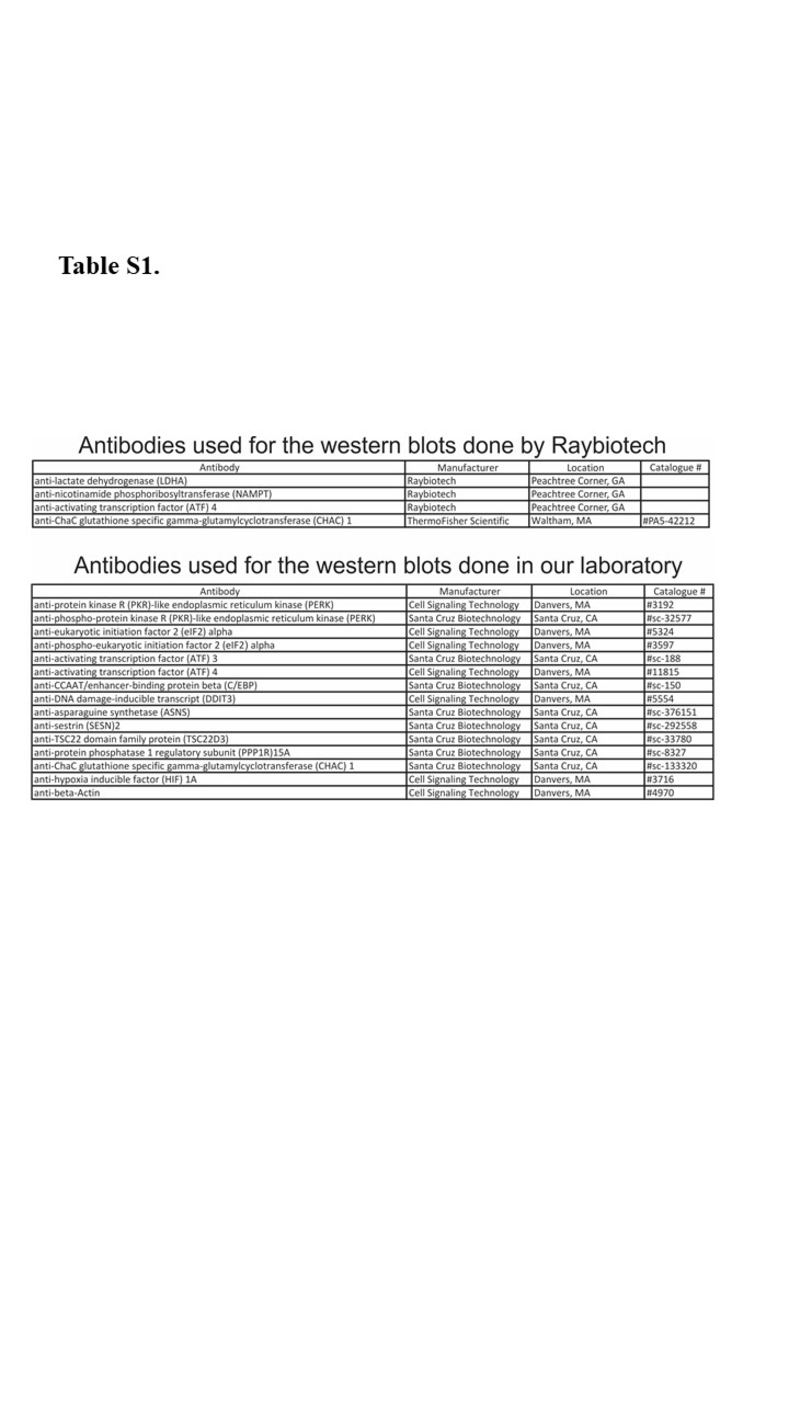

Supplement: S1 Table — (TIF) [file pone.0285536.s001.tif]

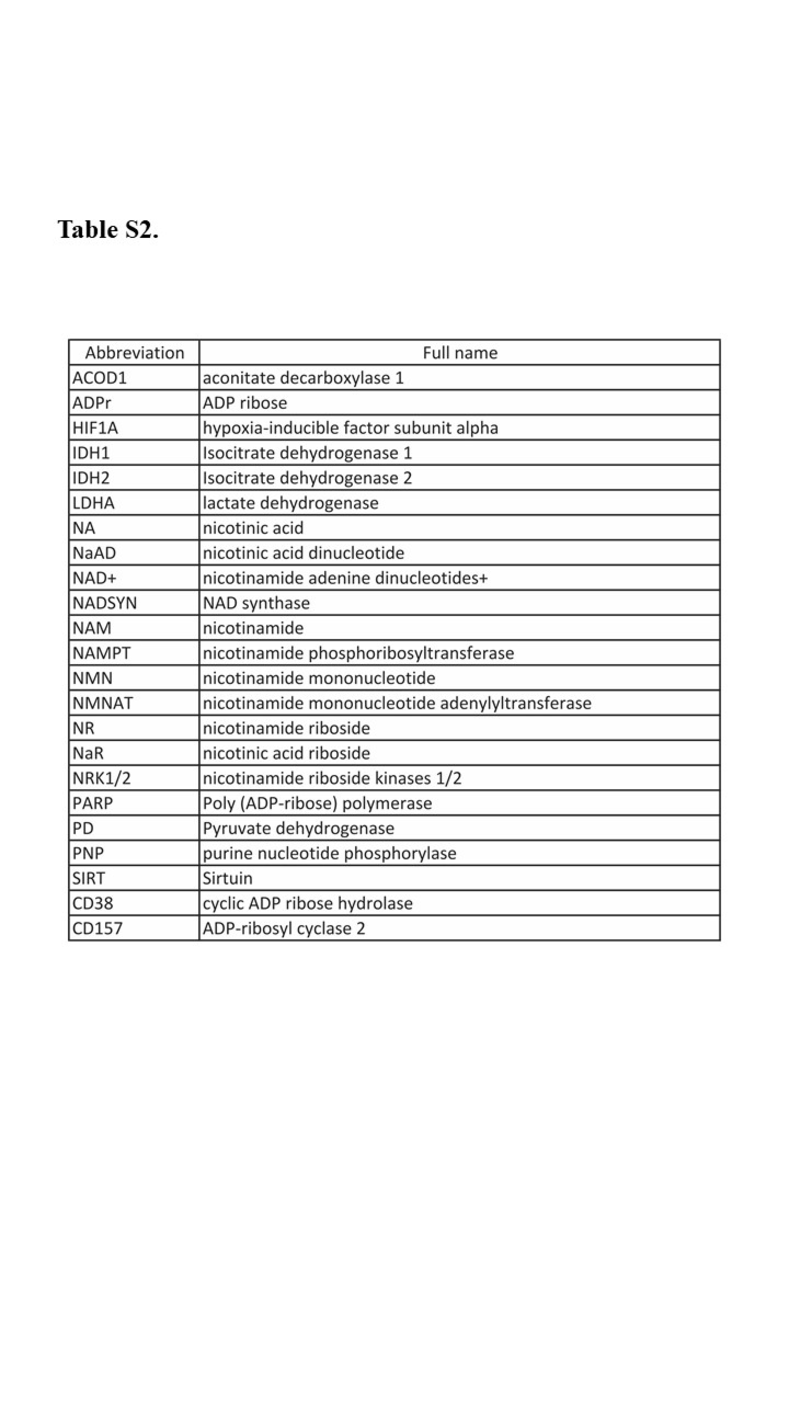

Supplement: S2 Table — (TIF) [file pone.0285536.s002.tif]

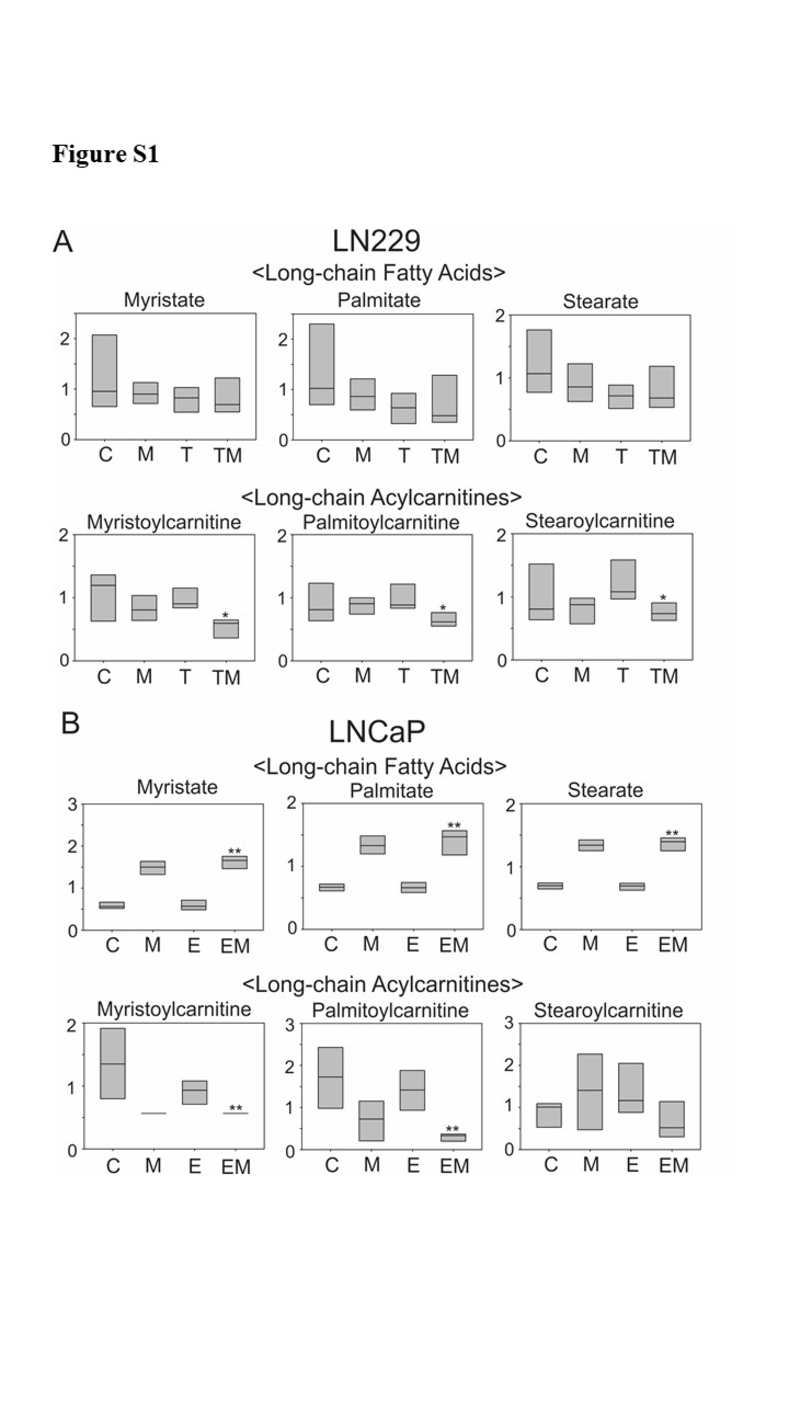

Supplement: S1 Fig — (TIF) [file pone.0285536.s003.tif]

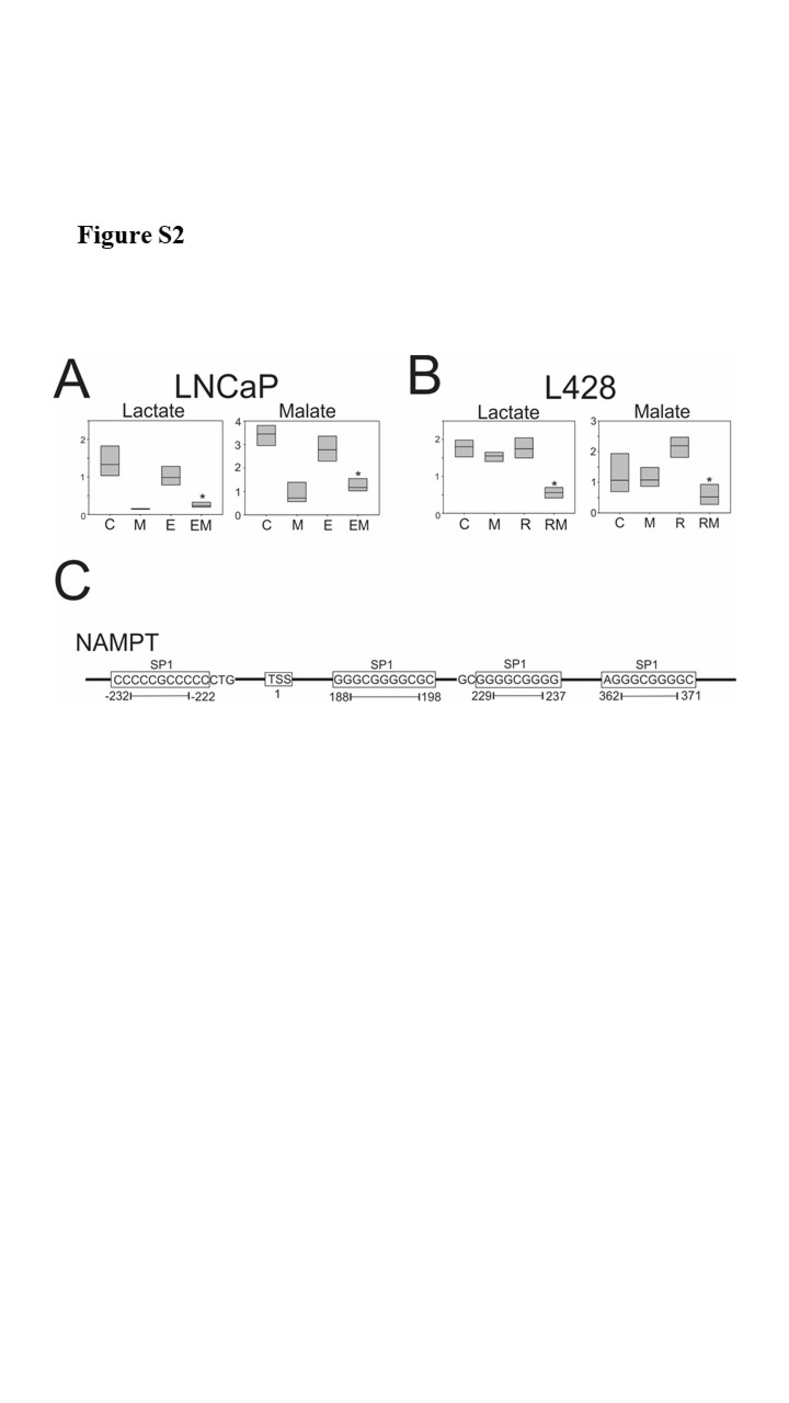

Supplement: S2 Fig — (TIF) [file pone.0285536.s004.tif]

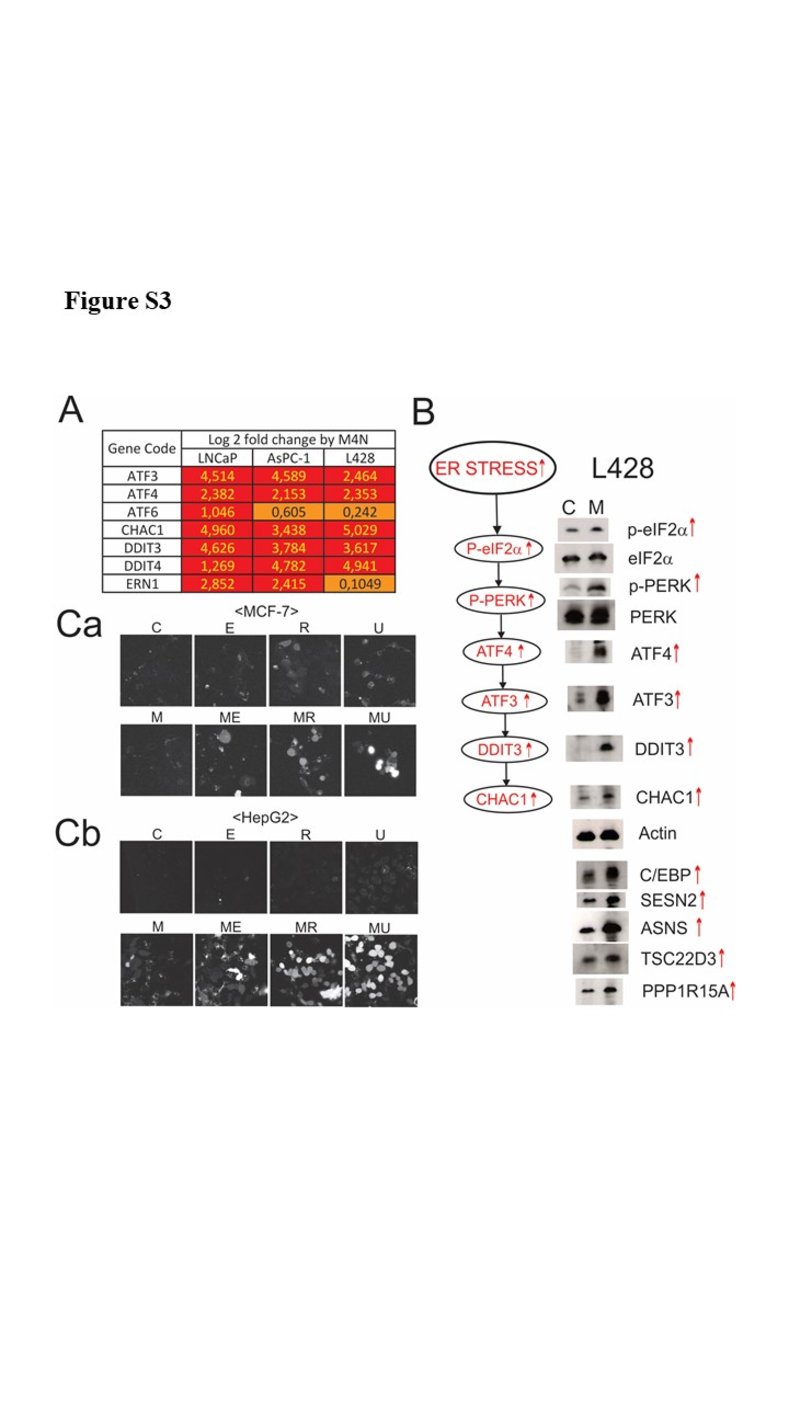

Supplement: S3 Fig — (TIF) [file pone.0285536.s005.tif]
