# Supplementary material for: Tetra-O-methyl-nordihydroguaiaretic acid inhibits energy metabolism and synergistically induces anticancer effects with temozolomide on LN229 glioblastoma tumors implanted in mice while preventing obesity in normal mice that consume high-fat diets
Source: PLoS One. 2023 May 25;18(5):e0285536. doi: 10.1371/journal.pone.0285536 (PMC10212158; doi:10.1371/journal.pone.0285536)
Supplement: S2 File — (DOCX) [file pone.0285536.s007.docx]

**Supporting Information**

**Long Title: Tetra-*O*-methyl-nordihydroguaiaretic acid inhibits energy metabolism and synergistically induces anticancer effects with temozolomide on LN229 glioblastoma tumors implanted in mice while preventing obesity in normal mice that consume high-fat diets**

**Short Title: M_4_N induces anticancer effect in combination with metabolism inhibition and prevents obesity**

Kotohiko Kimura^a^, Jong Ho Chun^a^, Yu-Ling Lin^b^, Yu-Chuan Liang^b^, Tiffany L. B. Jackson^a^, and Ru Chih C. Huang^a,c,^*

^a^Department of Biology, Johns Hopkins University, Baltimore, MD 21218-2685, USA

^b^Agricultural Biotechnology Research Center, Academia Sinica, Taipei, 11529, Taiwan, ROC

^c^Academician, Academia Sinica, Taipei 115, Taiwan, ROC

*Corresponding author

Ru Chih C. Huang, Ph.D., Department of Biology, Johns Hopkins University, 3400 N. Charles Street, Baltimore, MD 21218-2685, Tel: 410 516-5181; Fax: 410 516-5213; Email: [rhuang@jhu.edu](mailto:rhuang@jhu.edu)

**Table S1. A list of antibodies used for Western blotting.**


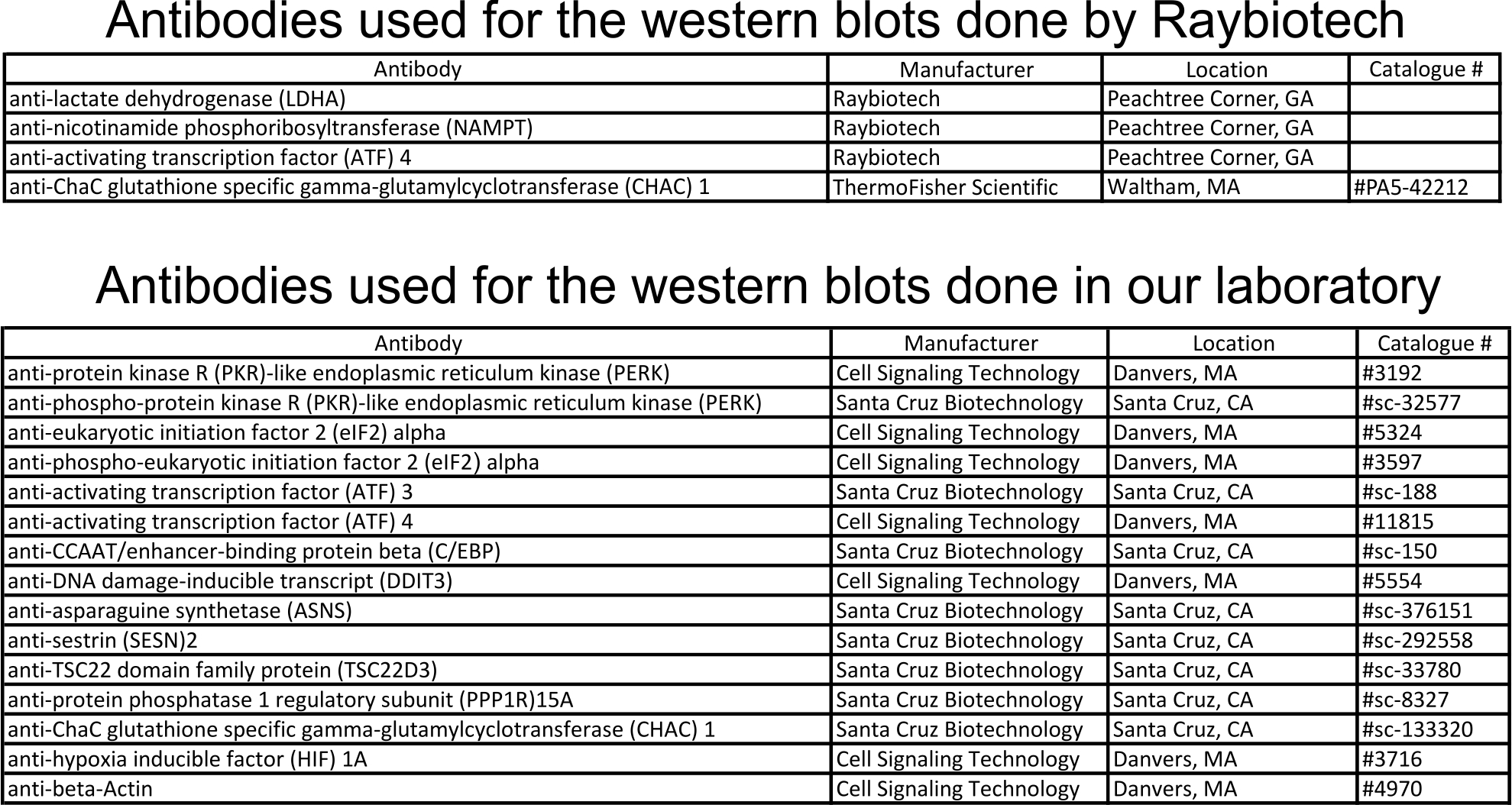


The antibodies are listed under two categories: antibodies used for the Western blots done by RayBiotech (Peachtree Corner, GA, USA) and the antibodies used for the Western blots done in our laboratory.

**Table S2. Abbreviation list.**

**
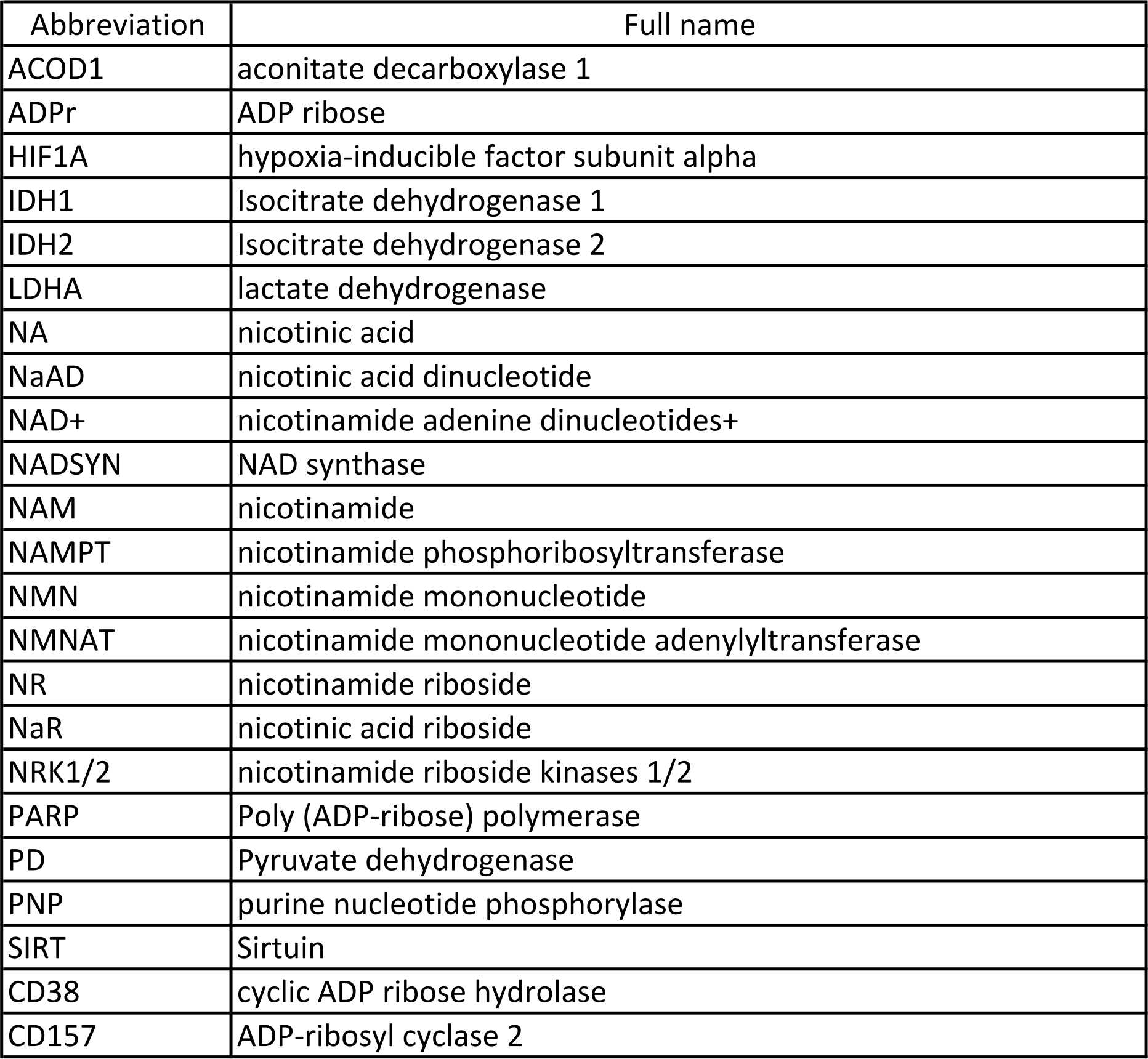
**


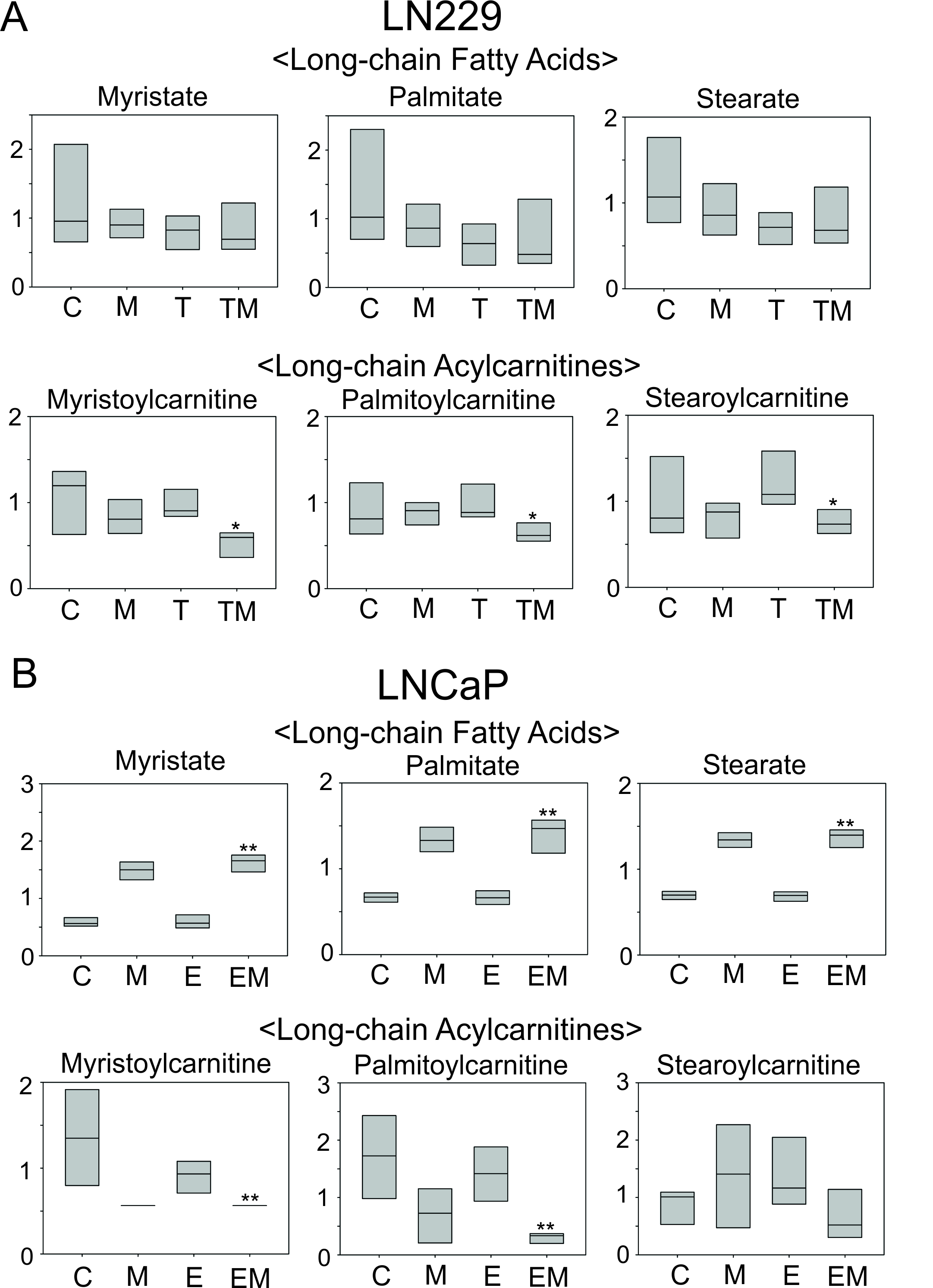
**Figure S1. M_4_N combination treatments suppressed long-chain acylcarnitine contents. (A)** The contents of long-chain fatty acylcarnitines were reduced in LN229 glioblastoma tumors implanted in xenograft mice treated with M_4_N and TMZ for 25 days. The box figures show the amounts of metabolites in the tumors treated with vehicle alone (C), M_4_N alone (M), TMZ alone (T) or TMZ+M_4_N (TM) on an arbitrary scale. The difference in the amounts of long-chain fatty acylcarnitines between the tumors treated with TMZ alone and those with TMZ+M_4_N was statistically significant by the Student’s *t*-test (*p<0.05). **(B)** The contents of long-chain fatty acylcarnitines were reduced while those of long-chain fatty acids were increased in LNCaP prostate cancer cells at 8 h after treatment with M_4_N or M_4_N+etoposide. LNCaP cells were treated with vehicle only, M_4_N (80 µM) only, etoposide (10 µM) only, or M_4_N (80 µM) plus etoposide (10 µM). The box figures show the amounts of metabolites in the tumors treated with vehicle alone (C), M_4_N alone (M), etoposide alone (E), or etoposide+M_4_N (EM) on an arbitrary scale. The difference in amount of long-chain fatty acylcarnitines or long-chain fatty acids between the tumors treated with etoposide alone and those with etoposide+M_4_N was statistically significant by the Student’s *t*-test (**: p<0.01). The data points are from tumors of five mice (A) or from five tissue cultures (B). The upper edge of each box represents a limit of the upper quartile, while the lower edge represents a limit of the lower quartile (A and B). The line in the middle of each box represents a median value (A and B).


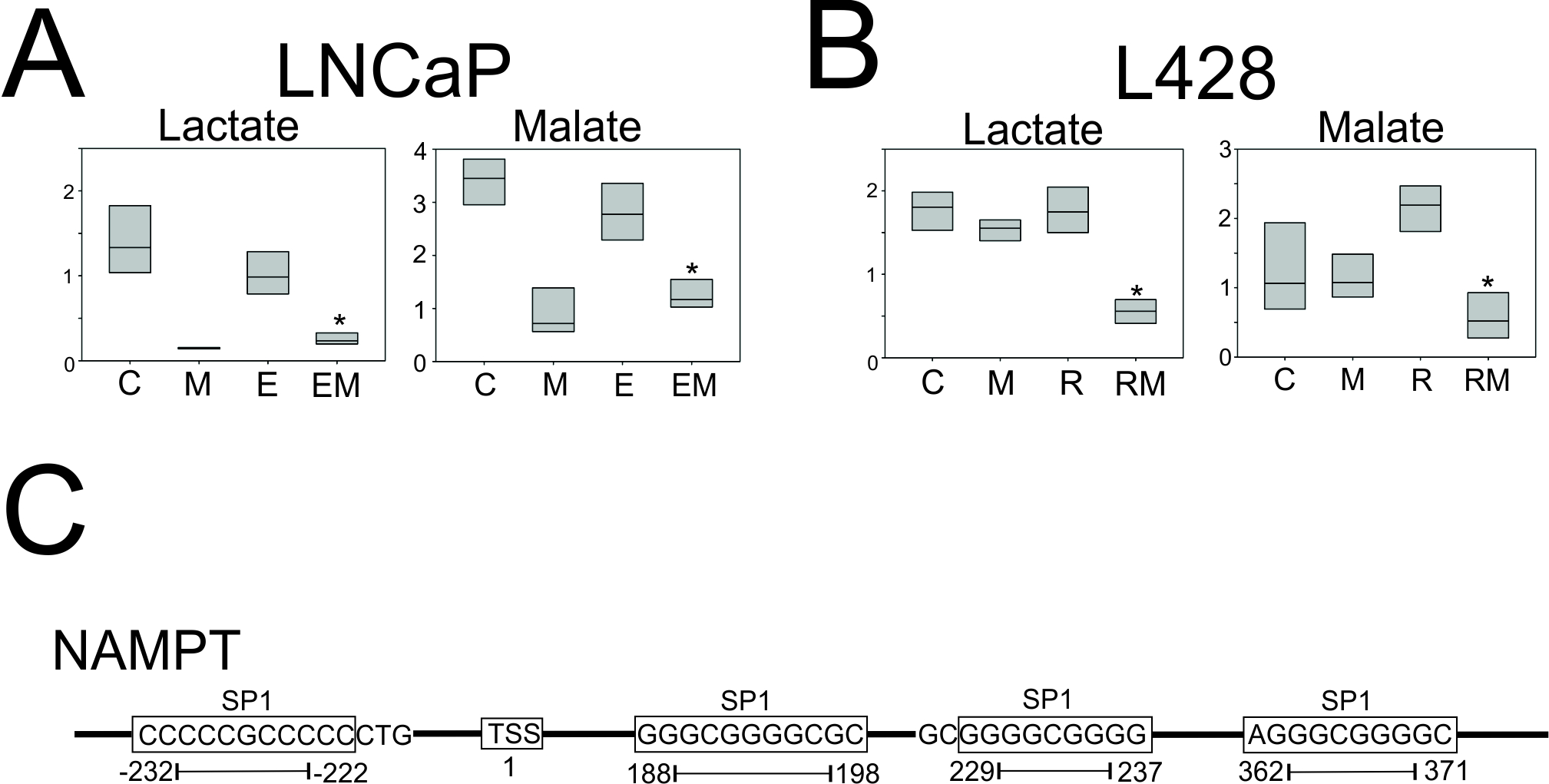


**Figure S2. Supplemental data about the effect of M_4_N on energy metabolism. (A and B) Effects of M_4_N combination treatments on energy metabolism (glycolysis and the tricarboxylic acid cycle) in cultured LNCaP prostate and L428 lymphatic cancers. (A)** Glycolysis and the TCA cycle were suppressed in cultured LNCaP cells at 8 h after treatment with M_4_N or M_4_N+etoposide. LNCaP cells were treated with vehicle only, M_4_N (80 µM) only, etoposide (10 µM) only, or M_4_N (80 µM) plus etoposide (10 µM). The box figures show the amounts of metabolites on an arbitrary scale. The difference in the amount of lactate or malate between the LNCaP tissue culture cells treated with etoposide alone and those with etoposide+M_4_N was statistically significant by Student’s *t*-test (*p<0.01). **(B)** Glycolysis and the TCA cycle were suppressed in cultured L428 cells at 8 h after treatment with M_4_N+rapamycin. L428 cells were treated with vehicle only, M_4_N (60 µM) only, rapamycin (10 µM) only, or M_4_N (60 µM) plus rapamycin (10 µM). The box figures showed the amounts of metabolites on an arbitrary scale. The amount of lactate or malate between the L428 tissue culture cells treated with rapamycin alone and those with rapamycin+M_4_N was statistically significant by the Student’s *t*-test (*: p<0.01). **(A and B)** The data points are from five different cell cultures. Control: (vehicle alone) (C), M_4_N alone (M), TMZ alone (T), TMZ+M_4_N (TM), etoposide alone (E), etoposide+M_4_N (EM), rapamycin alone (R), and rapamycin+M_4_N (RM). The upper edge of each box represents a limit of the upper quartile, whereas the lower edge represents a limit of the lower quartile. The line in the middle of each box represents a median value. **(C)** The promoter structures of the NAMPT gene. NAMPT gene contains four SP1 consensus sequences adjacent to transcription start site (TSS). The numbers indicate the locations of nucleotides relative to the TSS.


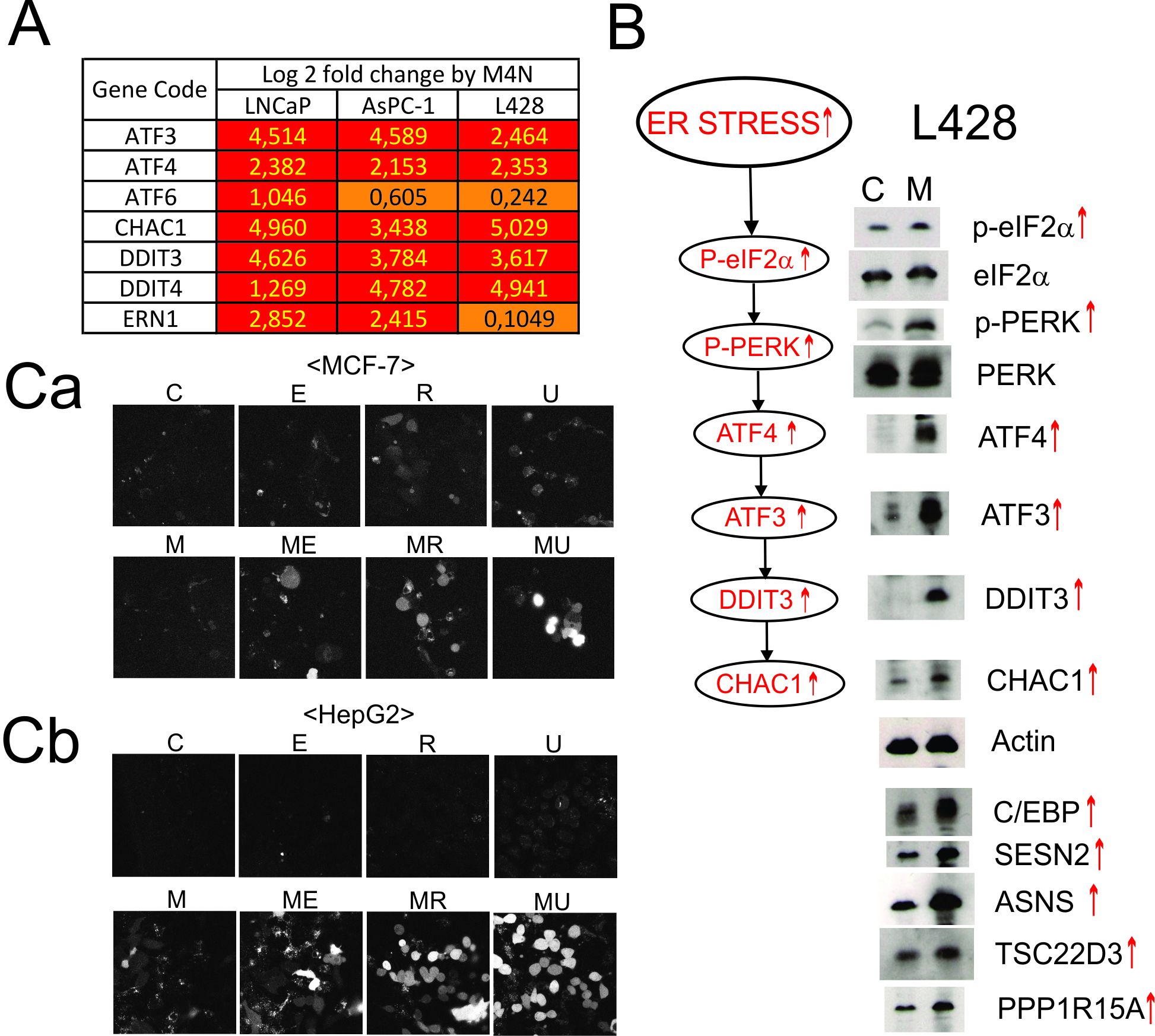


**Figure S3. M_4_N induced stress-related genes and synergistically generated reactive oxygen species in combination treatments. (A)** Stress-related genes were selected from the genes whose expression was significantly (p<0.05) modulated in common with all three cell lines (LNCaP prostate, AsPC-1 pancreatic, and L428 lymphatic cancer cells) by the treatment for 6 h with M_4_N (80 µM for LNCaP, 60 μM for AsPC1, and 60 μM for L428 cells) according to deep RNA-sequencing analysis. The column “log2 fold change by M_4_N” indicates the fold change in expression of each gene induced by M_4_N treatment on a log2 scale. The red color indicates a log2 fold change > 1.0; orange color indicates a log2 fold change between 0.0 and 1.0. The data points are from five tissue cultures. **(B)** M_4_N-activated stress-related signaling pathway in L428 cells. L428 cells were treated with M_4_N (60 µM) for 24 h. Western blotting was performed with antibodies against the proteins designated in the figure (phospho-protein kinase R-like endoplasmic reticulum kinase [PERK] [Thr^980^], PERK, phospho-eukaryotic translation initiation factor 2A [eIF2a] [Ser^51^], eIF2a, ATF4, ATF3, DDIT3, and CHAC1). Phospho-PERK [Thr^980^] and phospho-eIF2a [Ser^51^] are activated forms of PERK and eIF2a, respectively. The upper arrows indicate that M_4_N increased the expressions of these proteins. The descriptions on the left side illustrate the ATF4-ATF3-DDIT3-CHAC1 cascade mechanism. **(C)** M_4_N synergistically induced the production of ROS in combination with second anticancer drugs in human breast cancer MCF-7 cells (Ca). MCF-7 cells were treated with M_4_N (80 µM), etoposide (30 µM), rapamycin (30 µM), or UCN-01 (2 µM). ROS content was measured at 5 h after treatment. M_4_N also synergistically induced the production of ROS in combination with etoposide, rapamycin, or UCN-01 in human liver cancer HepG2 cells (Cb). HepG2 cells were treated with M_4_N (80 µM) in combination with etoposide (10 µM), rapamycin (20 µM), or UCN-01 (2 µM) and ROS contents were measured at 5 h after treatment. (Ca and Cb) C: control, E: etoposide, R: rapamycin, U: UCN-01, M: M_4_N, ME: M_4_N+etoposide, MR: M_4_N+rapamycin, and MU: M_4_N+UCN-01.

**Expanded Material and Methods**

**Cell culture conditions.** AsPC-1 pancreatic cancer cells were cultured in Dulbecco’s modified Eagle medium (DMEM) supplemented with fetal bovine serum (FBS) (10%). GFP-labeled LN229 cells were maintained in DMEM supplemented with FBS (5%). HepG2 cells were cultured in MEM supplemented with FBS (10%). HeLa cells were cultured in DMEM supplemented with FBS (10%) in 5% CO_2_ and 95% air for normoxia experiments. HL-1 cells were cultured in Claycomb medium (Sigma, Saint Louis, MO) supplemented with FBS (10%), norepinephrine (0.1 mM), and l-glutamine (2 mM). All the tissue culture media contained penicillin (100 units/mL) and streptomycin (100 μg/ml).

**Animals used for collecting LN229 tumor samples.** Female BALB/c nu mice aged 5 to 6 weeks were purchased from the National Laboratory Animal Center. All animals were subjected to health monitoring and maintained on a 12:12-h light:dark cycle with controlled temperature (22 ± 2°C) and humidity (55 ±10%) in a specific pathogen-free animal facility. They were acclimatized with free access to a standard rodent diet (5010; Lab Diet, St. Louis, MO, USA) and water. All procedures were approved by the Institutional Animal Care and Use Committee and followed the guidelines for the Use of Laboratory Animals (National Academy Press, Washington, DC, USA).

**Vitamin EM formulation.** This formulation was used as vehicle for the drug treatment of LN229 tumor-bearing xenograft mice. Procedures for EM formulation were previously described [S1]. Briefly, approximately 440 mg of a mixture of D-α-Tocopherol (vitamin E) polyethylene glycol 1000 succinate (BioXtra, water-soluble vitamin E conjugate; Millipore Sigma - SKU: 57668-5G) was heated to approximately 50°C. Approximately 5 mL oMiglyol 812N (IOI Oleo GmbH, Hamburg, Germany) was added to this mixture and stirred for approximately 15 min at room temperature. If solids were found in the formulation, gentle warming was performed (up to approximately 37–40°C) until the complete dissolution occurred. The EM oral formulation was stored at room temperature. On the day of the experiment, the control formulation was stirred at approximately 37–40°C for at least 30 min before dosing.

**Surgical implantation of HepG2 and AsPC1 tumors.** Procedure studied the effects of M_4_N on the life span of tumor-bearing xenograft mice. Cancer cells growing subconfluently were collected into the tissue culture medium without FBS and antibiotics. The cell concentration was adjusted with the same medium. After 50 µL of medium containing about 2 × 10^6^ cells had been mixed with the same volume of Matrigel (BD Science, Bedford, MA, USA), the combined solution was injected into the skin of nude mice. The tumor tissue growing subcutaneously was used for surgical implantation. The tumor tissue extracted from the skin was excised into pieces of about 2 mm diameter. After nude mice were anesthetized with 2,2,2-tribromoethanol (Aldrich Chemical Co. Inc., Milwaukee, WI, USA), a tumor tissue piece was implanted on the back of mice. Three days after the operation, intravenous injections of drugs were started. The numbers of mice that died after tumor implantation were counted for each group on a daily basis. After 180 days, all surviving mice were humanely euthanized.

**Drug injection methods and schedules for animal studies.** Animal studies were done to examine the effects of M_4_N+sorafenib combination treatments on HepG2 and AsPC1 tumor-bearing xenograft mice. Solutions for treatment were sorafenib (8.9 mg/mL) and M_4_N (10 mg/mL), which were formulated independently in CPE 25/30 vehicle [S2] for treatment where each mouse received 0.1 mL dose/day. For drug combinations (M_4_N/sorafenib), sorafenib powder was further added to M_4_N in CPE 25/30 to make a final concentration of M_4_N (10 mg/mL) with sorafenib (8.9 mg/mL) in CPE 25/30 formulation. These drugs were intravenously injected into the tail vein of mice at a daily dose of 0.1 mL per mouse. The drug injections were performed once every day, from day 3 until day 31, after tumor inoculation. The drugs were then injected once a week. The number of mice in each experimental group was either five or six for the control, sorafenib alone, M_4_N alone, and M_4_N+sorafenib groups. All animals used for the experiments were represented for the data without any exclusion. Mice were kept under a 12-h light/dark cycle in 23°C conditions in a well-controlled animal facility. Protocols used in this study were approved by the Institutional Animal Care and Use Committee at Johns Hopkins University (Baltimore, MD, USA).

**Animals used for the assessment of body weight after the consumption of high-fat diets containing the M_4_N drug.** Eight-week-old male C57BL|6J mice (The Jackson Laboratory, Bar Harbor, ME, USA) were used to model diet-induced obesity. They were chosen because their induced hepatic steatosis and hepatic insulin resistance are pathologically similar to the human disease [S3]. All mice were managed under the guidelines of the Institutional Animal Care and Use Committee at Johns Hopkins University. The housing and dietary methods used were similar to those performed by Murase et al. [S4]. Mice were kept under a 12-h light/dark cycle in 23°C conditions in a well-controlled animal facility.

**Diets used for the assessment of M_4_N-containing foods on weight.** The two diets consisted of the following ingredients: a) HF diet: 120 g basal mix, 5 mL water, and 45 mL corn oil; b) HFM diet: 120 g basal mix, 5 mL water, and 45 mL corn oil with 25 mg/mL M_4_N (6.83 mg M_4_N per g of food). The basal mix (Harlan Laboratories Inc., Frederick, MD, USA) was composed of casein, L-cystine, corn starch, maltodextrin, sucrose, cellulose, mineral mix, vitamin mix, choline bitartrate, and a tert-butylhydroquinone antioxidant. The nutritional composition of the basal mix (by weight) was 64.6% carbohydrates, 19.0% protein, and 0.2% fat. Food was made periodically in bulk per diet, according to the need of each group. Mice were given *ad libitum* access to food and water. Unused food was stored at 4°C.

**Organ collection.** Procedure to measure M_4_N contents in mice that ate HFM diets. On the final day of the experiment, at the age of 16 weeks, HF and HFM mice were humanly euthanized and dissected to retrieve the fat deposited at specific sites of the body. Fat pads were used as an indicator of fat metabolism and collected from the retroperitoneal, mesenteric, subcutaneous, and epididymal regions of the body. Fat pads were preserved using liquid nitrogen. Blood and small liver segments were stored separately for future analysis. The brain, liver, kidneys, lungs, gastrointestinal system, and pancreas were also removed and preserved using liquid nitrogen. These organs were stored by organ group, and pooled by either HF or HFM.

**TLC.** TLC was used for the measurement of M_4_N contents in the organs from mice that ate HFM diets. It was performed to see if visible levels of M_4_N could be detected through an extraction process. A sample of brain, liver, kidney, lung, gastrointestinal system, or pancreas from either HD- or HFD-fed mice was weighed, ground to powder, and dried. Then samples were extracted in a methanol:acetonitrile:acetone (MeOH:ACN:acetone;1:1:1) solvent using a volume five times the original weight of the tissue, and this mixture was incubated at room temperature overnight. The mixture was then centrifuged at 8000 rpm for 30 min at 0°C, and the supernatant was saved for TLC. Then the pellet was re-extracted using the same volume of MeOH:ACN:acetone, incubated at room temperature overnight, and centrifuged at 8000 rpm for 30 min at 0°C. The supernatant was again saved for TLC. TLC was run using a hexane:ethyl acetate (3:1) eluent. Once dry, silicon plates were sprayed with a 5% sulfuric acid in ethanol solution, and then heated at 150°C for visualization. HF and HFD extracted tissues were run alongside 300, 150, 75, and 37.5 ng M_4_N standards. M_4_N standards were created by first dissolving 4 mg M_4_N in 10 mL of MeOH:ACN:acetone (1:1:1), and then making serial dilutions using MeOH:ACN:acetone (1:1:1). The amount of M_4_N in the organs was estimated by comparing the intensity of spots from the extracted organ samples with that of M_4_N standards.

**HPLC-MS/MS.** This procedure was done to identify and quantify M_4_N levels in the organs from mice that ate HFM. Brain, liver, kidney, lung, gastrointestinal tract, pancreas, or fat samples were weighed, ground to powder, and dried. Then samples were extracted using 5 mL methanol:acetonitrile:acetone (MeoH:ACN:acetone; 1:1:1) solvent. The solution was mixed and kept at -20°C for 20 min. Then samples were centrifuged at 13000 rpm for 10 min at 4°C. The supernatant was removed and centrifuged at 13,000 rpm for 10 min at 4°C. The supernatant was again removed, and the sample was dried at room temperature. Then 500 µL acetonitrile was added to the recovered sample, and 10 µL of this solution was used for HPLC-MS/MS. The following dilutions had to occur to perform the analysis: gastrointestinal tract 100x, kidneys 10x, lungs 10x, and fat pad 10 to 100x. For HPLC conditions, the column was the Phenornenex Luna 3 µL C18(2) 150 x 2.0 mm with an injection volume of 10 µL. The flow rate was 0.2 mL/min and the MS run time was 70 min. The solvent was composed of varying ratios of 0.1% formic acid (FA) in water (H_2_O) and 0.1% FA in MeOH. The initial solvent composition was 10% FA and MeOH at 0 min, and then was increased linearly to 100% FA and MeOH at 60 min. The solvent was kept at 100% FA and MeOH from 60 to 70 min.

**Western blot analyses.** After cells had been grown in 25 mm^2^ flasks and treated with reagents, they were washed with PBS (137 mM NaCl, 2.7 mM KCl, Na_2_HPO_4_ 10 mM, and KH_2_PO_4_ 1.8 mM) three times and suspended in RIPA buffer (150 mM NaCl, 50 mM Tris-HCl [pH 8.0], 0.1% sodium dodecyl sulfate [SDS], 1% NP40, and 0.5% deoxycholate) supplemented with protease inhibitor cocktail (Calbiochem, San Diego, CA, USA). The viscous cell lysate was sonicated for 5–10 s with the Vibra-Cell sonicator (Sonics and Materials) with the microtip set at 40% output. The sample volumes were adjusted by the total protein amount. The protein assay was performed with the Bio-Rad Protein Assay Kit (Bio-Rad Laboratories Inc., Hercules, CA, USA). The samples were resolved by standard SDS-polyacrylamide gel electrophoresis and transferred to Hybond-ECL nitrocellulose membrane (Amersham Biosciences, Bjorkgatan, Sweden) using a semi-dry electroblot apparatus. The membranes were blocked with skim milk and incubated with primary antibodies at 4°C overnight and then with secondary antibody conjugated with horseradish peroxidase at room temperature for 2 h. The signals were detected by western blot chemiluminescence reagent plus (New England Nuclear Life Science Products, Boston, MA, USA). Antibodies are listed in the supplement.

**Northern blot analyses.** Total RNA was extracted from cells with Trizol Reagent (Invitrogen) and isolated according to the manufacturer’s protocol. Briefly, 24 μg total RNA was electrophoresed on formaldehyde-containing agarose gels. Resolved RNA was transferred to Nytran SPC 0.45 nylon transfer membranes (Whatman) by an upward capillary method and UV cross-linked to the membranes. Hybridization was carried out at 42°C in a hybridization solution containing 50% formamide, using random primed ^32^P-labeled probes of cDNAs specific for HIF1A. To assess variation in loading and transfer of RNA, the blots were stripped and reprobed with ^32^P-labeled β-actin cDNA. Levels of gene-specific mRNA were revealed by autoradiography.

**MTT assay.** LN229 cells were plated in 96-well microplates. After 48 h, M_4_N and TMZ were prepared at 4 mM stock solutions in DMSO (the chemicals were dissolved directly in DMSO) and added to the tissue culture medium in the wells. At 48 h after drug treatment, plates were removed from the incubator and 10 μL MTS (Promega CellTiter 96® AQueous non-radioactive cell proliferation assay) was added directly to each well. Plates were returned to the incubator for 2–4 h. The measurement of absorbance was carried out at 490 nm using the Power Wave 200 Microplate Scanning Spectrometer.

**Supplemental Materials and Methods**

**Reagents.** Etoposide, rapamycin, and UCN-01 were purchased from Millipore Sigma (St. Louis, MO, USA). The antibodies used in the experiments are described in the supplement (Table S1).

**Cell Culture.** LNCaP prostate cancer and MCF-7 breast cancer lines were purchased from American Type Culture Collection (Manassas, VA, USA). LNCaP cells were cultured in RPMI 1640 medium supplemented with glucose (14 mM), pyruvate (1 mM), and 10% FBS. MCF-7 cells were cultured in DMEM supplemented with FBS (10%). L428 Hodgkin lymphoma cell line was purchased from Leibniz Institute DSMZ-German Collection of Microorganisms and Cell Cultures (Braunschweig, Germany). L428 cells were cultured in RPMI 1640 medium supplemented with 10% FBS.

**Analysis of cell metabolites.** LNCaP cells were treated with M_4_N (80 µM) and/or etoposide (10 µM) while L428 cells were treated with M_4_N (60 µM) and/or rapamycin (10 µM). After treatment for 8 h, the cells were quickly washed with phosphate-buffered saline, and then immediately frozen with dry ice/ethanol. The samples were analyzed by Metabolon Inc. (Durham, NC, USA).

**Deep RNA-sequencing analysis.** LNCaP, and AsPC-1 cells were cultured in T75 flasks and treated with M_4_N (80 µM) for 6 h. RNA was extracted from the cells by Trizol reagent (Invitrogen, Carlsbad, CA) and was cleaned by RNeasy kit (Qiagen, Valencia, CA, USA), according to the manufacturer’s protocol. The deep RNA-sequencing analysis was done at the Deep Sequencing and Microarray Core, Johns Hopkins Medical Institutes. To analyze RNA sequence data, reads were first mapped to the human genome (hg19) using TopHat 1.4 [S5] and differential expression was detected using the Cuffdiff module in Cufflinks [S6], and Ensembl transcriptome as a guide.

**Reactive oxygen species production assay.** The reactive oxygen species (ROS) assay was performed using Image-iT LIVE Green Reactive Oxygen Species Detection Kit (I36007; Invitrogen, Grand Island, NY, USA), according to the manufacturer’s protocol. The cells were cultured in 6-well microwell dishes with coverslips for 48 h and further incubated in the medium containing M_4_N (80 µM) for another 9 to 10 h. After the cells had been washed with warm HBSS/Ca/Mg buffer (Gibco #14025-092; Invitrogen), the cells were incubated in HBSS/Ca/Mg containing 25 µM 5-(and-6)-carboxy-2′,7′-dichlorodihydrofluorescein diacetate (carboxy-H_2_DCFDA) at 37°C in the dark for 30 min. Then the cells were washed very gently with HBSS/Ca/Mg buffer three times. The cells were observed through B29/Zeiss LSM 510 META laser confocal microscope (Carl Zeiss, Oberkochen, Germany). The cell images were captured with a 488 nm argon-ion laser, because the oxidation product of carboxy-H_2_DCFDA has excitation/emission maxima of approximately 495/529 nm.

**Computer analysis of gene promoters.** The promoter analysis was performed using Transfac 2.0 (geneXplain, Wolfenbüttel, Germany).

**Supplemental References**

S1. Huang RC, Chun J, Lin Y, Liang Y, Liao KW, Jackson, T, Lai C, Mold D. Formulations of terameprocol and temozolomide and their use in stimulation of humoral immunity in tumors. March 2021. Publication Number: WO/2021/108601.

S2. Lopez RA, Goodman AB, Rhodes M, Blomberg JA, Heller J. The anticancer activity of the transcription inhibitor terameprocol (meso-tetra-O-methyl nordihydroguaiaretic acid) formulated for systemic administration. Anticancer Drugs. 2007; 18: 933-9.

S3. Buettner R, Scholmerich J, Bollheimer L. High-fat Diets: Modeling the Metabolic Disorders of Human Obesity in Rodernts. Obesity. 2007; 15: 798-808.

S4. Murase T, Misawa K, Minegishi Y, Aoki M, Ominami H, Suzuki Y, Shibuya Y, Hase T. Coffee polyphenols suppress diet-induced body fat accumulation by downregulating SREBP-1c and related molecules in C57BL/6J mice. Am. J. Physiol. Endocrinol. Metab. 2011; 300: E122-33. doi:10.1152/ajpendo.00441.2010. Epub 2010 Oct 13. PMID: 20943752.

S5. Trapnell C, Pachter L, Salzberg SL. TopHat: discovering splice junctions with RNA-Seq. Bioinformatics. 2009; 25: 1105-11.

S6. Trapnell C. Williams BA, Pertea G, Mortazavi A, Kwan G, van Baren MJ. et al. Transcript assembly and quantification by RNA-Seq reveals unannotated transcripts and isoform switching during cell differentiation. Nature Biotechnol. 2010; 28: 511-5. doi:10.1038/nbt. 1621. Epub 2010 May 2.
